# Role of *NDE1* in the Development and Evolution of the Gyrified Cortex

**DOI:** 10.3389/fnins.2020.617513

**Published:** 2020-12-18

**Authors:** Jaseph Soto-Perez, Marybeth Baumgartner, Rahul N. Kanadia

**Affiliations:** ^1^Department of Physiology and Neurobiology, University of Connecticut, Storrs, CT, United States; ^2^Department of Genetics, Yale School of Medicine, New Haven, CT, United States; ^3^Institute for Systems Genomics, University of Connecticut, Storrs, CT, United States

**Keywords:** *NDE1*, microcephaly, interkinetic nuclear migration, lissencephaly-4, evolution, cortical development, gyrification

## Abstract

An expanded cortex is a hallmark of human neurodevelopment and endows increased cognitive capabilities. Recent work has shown that the cell cycle-related gene *NDE1* is essential for proper cortical development. Patients who have mutations in *NDE1* exhibit congenital microcephaly as a primary phenotype. At the cellular level, *NDE1* is essential for interkinetic nuclear migration and mitosis of radial glial cells, which translates to an indispensable role in neurodevelopment. The nuclear migration function of *NDE1* is well conserved across Opisthokonta. In mammals, multiple isoforms containing alternate terminal exons, which influence the functionality of *NDE1*, have been reported. It has been noted that the pattern of terminal exon usage mirrors patterns of cortical complexity in mammals. To provide context to these findings, here, we provide a comprehensive review of the literature regarding *NDE1*, its molecular biology and physiological relevance at the cellular and organismal levels. In particular, we outline the potential roles of *NDE1* in progenitor cell behavior and explore the spectrum of *NDE1* pathogenic variants. Moreover, we assessed the evolutionary conservation of *NDE1* and interrogated whether the usage of alternative terminal exons is characteristic of species with gyrencephalic cortices. We found that gyrencephalic species are more likely to express transcripts that use the human-associated terminal exon, whereas lissencephalic species tend to express transcripts that use the mouse-associated terminal exon. Among gyrencephalic species, the human-associated terminal exon was preferentially expressed by those with a high order of gyrification. These findings underscore phylogenetic relationships between the preferential usage of *NDE1* terminal exon and high-order gyrification, which provide insight into cortical evolution underlying high-order brain functions.

## Introduction

In mammalian evolution, the brain has undergone significant change. Particularly in the human lineage, the cerebral cortex has vastly expanded, which underlies our self-awareness, increased intellectual capacity, and other higher-order executive functions (Geschwind and Rakic, 2013). As a result, the human brain has a complex, convoluted architecture. Cortical architecture varies widely across mammals. In some species, like humans, the cortex is folded in on itself, due to a substantial increase in the cortical area relative to total brain size, in a process called gyrification (Ronan and Fletcher, 2015). In other species, the surface of the cerebrum is smooth or lissencephalic. The extent to which mammalian brains form gyri can be quantified using the gyrification index (GI), which allows researchers to rank species by their degree of cortical folding (Zilles et al., 1988). Humans have a particularly high GI value, which has been suggested to play into our higher cognitive functions (Fjell et al., 2013).

Expansion of the cortex and gyrification emerged from changes in brain development, which requires tight coordination of proliferation and differentiation within a limited gestational window. For instance, experimental and cross-species comparative studies indicate that extension of the proliferative period early in cortical development, or lengthening of the neurogenic period itself, can drive cortical expansion (Finlay and Darlington, 1995; Geschwind and Rakic, 2013; Mora-Bermúdez et al., 2016; Benito-Kwiecinski et al., 2020; Eze et al., 2020; Stepien et al., 2020). The precise mechanisms underlying cortical expansion are complex, involving both the regulation of progenitor cell division and species-specific heterogeneity of progenitor populations (Reillo et al., 2010; Geschwind and Rakic, 2013; Borrell and Götz, 2014; Kalebic and Huttner, 2020). Specifically, neuroepithelial cells (NECs) and, later, radial glial cells (RGCs) must undergo proliferative divisions to adequately expand the progenitor cell pool (Jiang and Nardelli, 2016). In addition to self-amplification, RGCs can undergo differentiative divisions to produce one RGC and either a neuron or another progenitor cell type, such as an intermediate progenitor cell (IPC). These IPCs have a limited capacity to self-amplify, instead undergoing symmetric differentiative divisions to produce many neurons (Noctor et al., 2004; Kriegstein et al., 2006). In humans and other gyrencephalic species, RGCs are classified into two broad categories: (i) ventricular/apical RGCs (vRGCs/aRGCs), which primarily divide to produce RGCs of both types, and (ii) outer/basal RGCs (oRGCs/bRGCs), a basally located progenitor pool that undergoes self-amplification. These RGC types can be further subdivided by their morphology and molecular signatures (Betizeau et al., 2013; Jiang and Nardelli, 2016; Kalebic and Huttner, 2020; Li et al., 2020; Matsumoto et al., 2020). In gyrencephalic species, the oRGC pool is large enough to represent a distinct cellular layer of the developing cortex, known as the outer subventricular zone (OSVZ), which has been correlated with cortical complexity and folding (Dehay and Kennedy, 2007; Reillo et al., 2010; Borrell and Götz, 2014; Llinares-Benadero and Borrell, 2019). As a result, the final size of the cortex is highly dependent on vRGC and oRGC behavior and the balance between self-amplification versus differentiative division in these populations. This is best exemplified by the human brain, where both the timeframe of progenitor cell self-amplification and the size of the oRGC population are greatly expanded (Hansen et al., 2010; Geschwind and Rakic, 2013).

Progenitor cell cycle and behavior are controlled by complex developmental gene networks, in which small disturbances, such as single-gene mutations, can cascade into neurodevelopmental disorders, such as microcephaly (Cox et al., 2006). This is the case for multiple mutations in the gene *NDE1* (Alkuraya et al., 2011; Bakircioglu et al., 2011). *NDE1* is a highly conserved gene that is involved in interkinetic nuclear migration (INM) and mitosis. INM is a fundamental behavior in the cell cycle progression of RGC and NEC progenitors that ensures these populations expand appropriately (Jiang and Nardelli, 2016). As such, clinical cases of patients with mutations in *NDE1* as well as experimental data from mouse models suggest that disrupting *NDE1* results in a smaller RGC pool, likely due to its roles in cell cycle progression and progenitor cell behavior (Feng and Walsh, 2004; Pawlisz et al., 2008; Alkuraya et al., 2011; Bakircioglu et al., 2011; Guven et al., 2012; Abdel-Hamid et al., 2019).

Given its evolutionary conservation and relevance in cortical development, previous reports have postulated that evolutionary changes in *NDE1* expression may have contributed to observable differences in cortical size and complexity across species (Mosca et al., 2017; Monda and Cheeseman, 2018). In particular, Monda and Cheeseman have proposed that human-specific isoform expression may contribute to brain development differently than the mouse-specific isoform. Currently, there is no definitive evidence that these species-specific isoforms differentially influence cortical development. Moreover, no links have been made between the degree of brain gyrification and the emergence of *NDE1* species-specific isoforms.

In this review, *NDE1* is discussed as an important cell cycle-related gene that functions as a crucial determinant of cortical development. For this purpose, here, we evaluate the known functions of *NDE1* relative to established mechanisms involved in progenitor cell behavior and frame *NDE1* in the context of the pathophysiology associated with *NDE1* mutations. Additionally, we assess the evolutionary conservation of *NDE1* domains and whether species-specific changes in *NDE1* isoform expression are consistent with evolutionary expansions of cortical development. We propose that species-specific use of alternative transcription termination sites may be associated with the emergence of gyri in mammalian species.

## Identification of *Nde1*

*Nde1* was identified by Efimov and Morris (2000) while studying the mechanisms governing nuclear migration in fungi. In filamentous fungi such as *Aspergillus nidulans*, nuclear migration plays an essential part in growth and reproduction (Morris et al., 1995; Morris, 2000). Initial studies aimed at elucidating the mechanism responsible for nuclear migration were performed on *A. nidulans* Nud mutants, which exhibited disrupted nuclear migration (Morris et al., 1995). At the time, multiple gene mutations underlying these nuclear migration defects were known, such as those in the gene *NudF*. Studies had also shown that the *NudF* gene was homologous with the LIS1 protein in humans and that dynein was the motor that carried out nuclear migration in filamentous fungi (Morris, 1975; Xiang and Morris, 1999). While studying the protein–protein interaction involved in this mechanism, Efimov and Morris, identified a novel Nud mutation in a gene they named *NudE*. Moreover, their findings highlighted a functional interaction between the NudE and NudF proteins (Efimov and Morris, 2000). We now know that both NudE and NudF proteins have been evolutionarily conserved across Opisthokonta, including fungi and vertebrates. In humans, the ortholog of *NudE* is the gene *NDE1*.

*NDE1*/*NudE* has a paralog, known as *NudE* neurodevelopment protein 1 like 1 (*NDEL1*), which shares similar protein structure and function (Hayashi et al., 2005; Bradshaw et al., 2013). Like NDE1, NDEL1 can interact with LIS1 and dynein, and this interaction can form a motor complex that recapitulates some migratory functions in the absence of *NDE1* and the NDE1/LIS1/dynein complex (Doobin et al., 2016). Although NDEL1 is not the focal point of this review, it does also play an important and unique role in cortical development (Bradshaw et al., 2013; Bradshaw and Hayashi, 2016).

## *Nde1* Isoform and Protein Structure

**FIGURE 1:**
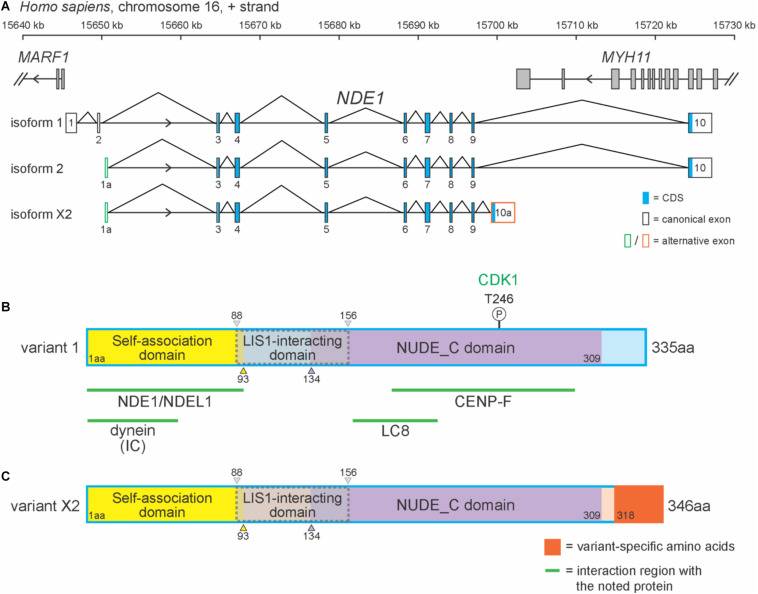
*NDE1* isoforms differ by exon usage and protein products. **(A)** Schematic of the human locus for *NDE1*, with three selected *NDE1* isoforms. Arrowheads indicate gene strand. Gray-filled boxes denote the exons of neighboring genes. **(B,C)** Schematics of **(B)** NDE1 protein variant 1, encoded by isoforms 1 and 2 and **(C)** NDE1 protein variant X2, encoded by isoform X2. Both are color-coded by self-associated domain (yellow), LIS1-interacting domain (gray with dotted gray outline), NUDE_C domain (purple), and sequence unassigned to a functional domain (blue or peach, respectively). Variant X2-specific sequence is shown in orange. Numbers indicate amino acid positions. Green lines and labels indicate binding partners and the regions of NDE1 with which they interact. One phosphorylation site is shown by a circled “P,” alongside the kinase (green) that mediates this modification.

In humans, *NDE1* is located on the forward strand of chromosome 16p13.11 and flanked by *MARF1* and *MYH11* (Figure 1A). *NDE1* has 15 isoforms, most of which (13) are predicted to exist by the National Center for Biotechnology Information (NCBI) algorithms. One of these predicted isoforms, isoform X2, is homologous to the canonical mouse *Nde1* transcript, which has been referenced in studies of species-specific *Nde1* isoforms in the literature. Here, we will be focusing on this predicted isoform alongside *NDE1* isoforms 1 and 2 (Figure 1A). Isoform 1 consists of 10 exons, whereas isoforms 2 and X2 contain 9 exons each (Figure 1A). Differences between these isoforms are found in the 5′ and 3′ untranslated regions. The terminal exon 10, found in both isoforms 1 and 2, overlaps with the 3′ neighboring gene *MYH11* (Figure 1A). Isoforms 1 and 2 also share the same open reading frame (exons 3–10). Isoform X2 shares most of its open reading frame (exons 3–9) with isoforms 1 and 2, except for the last 2.1 kbp encoded by its unique terminal exon 10a (Figure 1A). As a result, these three isoforms encode two different protein products: the canonical protein (protein variant 1), encoded by isoforms 1 and 2, and protein variant X2, which is encoded by isoform X2 (Figures 1B,C).

In humans, the canonical NDE1 protein is 335 amino acids in length and primarily composed of alpha-helices. As a consensus among the literature, the canonical NDE1 protein is considered to have three main domains: (1) a self-association domain (from M1 to I93); (2) a LIS1 interaction domain (E88 to L156), which shares its first five amino acids with the self-association domain; and (3) a NUDE_C domain at its C terminus (S134–S309) (Figure 1B; Guven et al., 2012; Mosca et al., 2017). These domains are highly conserved across evolution, as will be discussed later in this review.

Protein variant X2 differs from the canonical NDE1 protein at its C-terminus. Protein variant X2 is also longer than protein variant 1, containing 346 residues (Figures 1B,C). These variants are homologous until the 317th amino acid, after which point the amino acid residues diverge. It has been suggested that the canonical variant and the transcripts that encode it are newly evolved, given that they are only found in specific species, including primates and dogs (Monda and Cheeseman, 2018). Moreover, Mosca et al. (2017) reported that the non-canonical variant in humans (protein variant X2) is more similar to the canonical variant in mice, with these isoforms being ∼90% identical.

## Cellular Functions of Nde1 and Its Binding Partners

The initial framework for NDE1 protein–protein interaction was established in *A. nidulans Nud* mutants (Morris, 1975; Xiang and Morris, 1999; Efimov and Morris, 2000). Subsequent work has revealed numerous interacting partners and, in some cases, their discrete protein-binding regions in NDE1 (Bradshaw et al., 2013). In general, the NDE1 protein forms part of a motor complex with dynein and LIS1 (Efimov and Morris, 2000; Feng and Walsh, 2004; Vergnolle and Taylor, 2007; Monda and Cheeseman, 2018). For NDE1 to be a part of this motor complex, it must dimerize with itself or with its paralog NDEL1 (Soares et al., 2012; Bradshaw and Hayashi, 2016). As part of the complex, NDE1 can interact with centromere protein F (CENP-F) at different subcellular localization to control nuclear migration, cell cycle progression, and mitosis (Vergnolle and Taylor, 2007; Bertipaglia et al., 2018; Doobin and Vallee, 2018). A graphical representation of the most prominent NDE1-binding partners and their interacting region in the NDE1 sequence are depicted in Figure 1B. These protein interactions allow NDE1 to regulate nuclear migration, cell cycle progression, and mitosis.

The motor complex formed by interactions among NDE1, LIS1, and dynein attaches to the nucleus and carries it along the microtubules (Xiang and Morris, 1999; Efimov and Morris, 2000; Reiner et al., 2011). Dynein is a multimeric complex that functions in transporting cargo from the plus (+) end of microtubules toward the negative (−) end and has essential functions in mitosis, such as positioning of the mitotic spindle (Vergnolle and Taylor, 2007; Kiyomitsu and Cheeseman, 2012). Dynein subunits bind to different locations on NDE1, with the interaction site of dynein intermediate chain located in the self-association domain (Żyłkiewicz et al., 2011), whereas dynein light chain (LC8) interacts with the NUDE-C domain (Figure 1B; Bradshaw et al., 2013). One model proposed by Soares et al. (2012) suggests that dimers can acquire a “back-bent” conformation, where a proline-rich flexible linker region allows the C-terminal to bend backward to interact with the N-terminal, which may bring together the two dynein-binding regions near. Cyclin-dependent kinase 1 (CDK1) phosphorylation of the NDE1 NUDE-C domain at residue T246 is believed to be required for the interaction between NDE1 and LIS1, and eliminating this residue arrests cells in the G2/M transition (Figure 1B; Alkuraya et al., 2011). The NDE1/LIS1 complex then interacts with dynein to form the NDE1/LIS1/dynein motor complex, where LIS1 is responsible in part for the attachment of dynein onto the (+) end of microtubules and the loading of cargo onto the dynein complex (Derewenda et al., 2007; Pawlisz et al., 2008).

Aside from its prominent role in nuclear migration, the NDE1/LIS1/dynein motor complex has been shown to function in mitosis. As the nuclear envelope breaks down and mitosis begins, the centrosome nucleates microtubules, which will interact with the kinetochores of the chromosomes, to form the mitotic spindle. During mitosis, NDE1 localizes to spindle microtubules and kinetochores. As the spindle apparatus nucleates microtubules, the NDE1/LIS1/dynein complex focuses these fibers onto the kinetochores of mitotic chromosomes, essentially organizing the mitotic spindle (Vergnolle and Taylor, 2007). The focusing of spindle microtubules to kinetochores occurs through the interaction of NDE1’s NUDE_C domain with the centrosomal protein CENP-F, which then recruits the motor complex to the kinetochores (Vergnolle and Taylor, 2007). Accordingly, the depletion of NDE1 results in the failure of microtubules to focus on kinetochores (Vergnolle and Taylor, 2007). The focusing of spindle fibers onto kinetochores is a preparatory process for chromosome segregation, implicating the NDE1/CENP-F interaction in this process. Studies have shown that depletion of CENP-F results in failure of NDE1 to localize to the kinetochores, failure of NDE1/LIS1/dynein motor complex recruitment, and impaired chromosome segregation (Bomont et al., 2005; Vergnolle and Taylor, 2007; Monda and Cheeseman, 2018).

**FIGURE 2:**
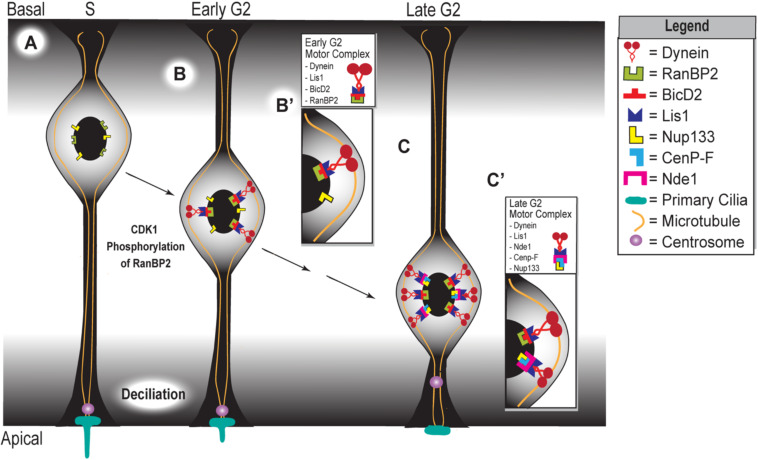
Apical movement during interkinetic nuclear migration depends on two distinct but complementary mechanisms. **(A)** Schematic representing the apical phase of interkinetic nuclear migration in a ventricular radial glial cell, with black arrows indicating the progression of both time and apical nuclear movement. Cell cycle phases are listed at the top of the schematic, which represents the embryonic developing cortex. Magnified insets show the composition of the two motor complexes that assemble during early **(B,B’)** and late **(C,C’)** G2 phases, which drive the early versus late phases of apical nuclear movement.

In addition to interacting with proteins that function in nuclear migration and mitosis, NDE1 can also regulate the functionality of the primary cilium. The primary cilium is a non-motile organelle that functions in signal transduction and regulates cell cycle progression in most mammalian cells (Pan and Snell, 2007; Tong et al., 2014; Bodle and Loboa, 2016). Specifically, the primary cilium is attached to the mother centriole of the centrosome, using it as its basal body during G1, S, and early G2 phases of the cell cycle (Pan and Snell, 2007; Bertipaglia et al., 2018). During mitosis, centrioles duplicate and the two centrosomes migrate toward opposite poles of the cell to form the mitotic spindle (Pan and Snell, 2007). For the mitotic spindle to form, the primary cilium must be resorbed so that the captive centriole is released (Figure 2). For this reason, the primary cilium is an influencer of cell cycle progression (Pan and Snell, 2007; Satir and Christensen, 2008). Recent evidence has highlighted NDE1 as a negative regulator of the primary cilium (Kim et al., 2011). It has been shown that NDE1’s NUDE-C domain inhibits ciliogenesis through its interaction with LC8 (Wynshaw-Boris, 2007; Kim et al., 2011; Spear and Erickson, 2012a). It has been proposed that NDE1-mediated deciliation is the result of NDE1 sequestering LC8 at the basal body, which prevents retrograde dynein from contributing to the formation and maintenance of the primary cilium (Kim et al., 2011). This interaction may be a driving force in primary cilium resorption and the subsequent liberation of the captive centriole, therefore regulating progenitor cell mitotic progression. Expression of NDE1 gradually increases at the beginning of the S phase, peaks at M-phase, and drops after mitosis (Kim et al., 2011). Given its role as an inhibitor of ciliogenesis, this NDE1 expression pattern fits the changes observed in the primary cilium throughout the cell cycle.

Although the physiological relevance of many NDE1 protein interactions has been well described in multiple model systems (Feng and Walsh, 2004; Pawlisz et al., 2008; Kim et al., 2011), other interactions have yet to be functionally characterized. In particular, a study by Monda and Cheeseman (2018) showed that NDE1 interacts with the S26 proteasome through its C-terminus and that this interaction is not required for the mitotic roles of NDE1 (Monda and Cheeseman, 2018). Although this implicates NDE1 in protein degradation pathways, the physiological relevance of this interaction remains unexplored (Monda and Cheeseman, 2018). Notably, NDE1 interacts with the 26S proteasome in an isoform-specific manner, where only the canonical human isoform (isoform 1 and 2), and not the mouse-associated isoform (isoform x2), is able to carry it out (Monda and Cheeseman, 2018). Species specificity for this interaction is the result of a divergence in the C-terminal of NDE1 among species. Taking into account NDE1’s prominent role in cortical development and the anatomical differences between the brains of humans and mice, it is possible that NDE1’s interaction with the proteasome differentially contributes to the neurodevelopment of lissencephalic and gyrencephalic species.

Although NDE1 has multiple protein-binding partners, the most well-characterized interactions are those that mediate homo- (NDE1/NDE1) and hetero- (NDE1/NDEL1) dimer formations (Efimov and Morris, 2000; Derewenda et al., 2007; Bradshaw et al., 2013; Monda and Cheeseman, 2018). Functional redundancy between NDE1 and NDEL1 allows both of these to form motor complexes with LIS1 and dynein, mediate nuclear migration, and interact with CENP-F (Vergnolle and Taylor, 2007; Doobin et al., 2016). However, despite NDEL1-binding CENP-F, it cannot compensate for the mitotic functions of NDE1 (Vergnolle and Taylor, 2007). This suggests that the interaction between the mitotic spindle microtubules and the kinetochore is only possible through NDE1, marking an essential function for this protein that cannot be recapitulated by NDEL1.

## *Nde1* in Development and Disease

Here, we discuss the role of *NDE1* in cell cycle regulation of neural progenitor cells, specifically its role in INM and how mutations in *NDE1* result in various human diseases in various human diseases that are often characterized by microcephaly (Alkuraya et al., 2011; Bakircioglu et al., 2011; Reiner et al., 2011; Guven et al., 2012; Tan et al., 2017; Abdel-Hamid et al., 2019).

## Regulation of Interkinetic Nuclear Migration in Neural Progenitor Cells During Cortical Development

In human cortical development, a monolayer of pseudostratified NECs gives rise to a multilayer, heterogeneous pool of progenitor cells, including vRGCs, oRGCs, and IPCs (Lui et al., 2011; Borrell, 2018). These progenitor cells are found in distinct locations in the developing cortex and have distinct morphologies. The vRGCs form the ventricular zone, which lines the ventricle. Like NECs, they have two processes that connect them to the ventricular and pial surfaces of the developing cortex, respectively (Jiang and Nardelli, 2016). In contrast, the oRGCs and IPCs are found in a more basal layer called the subventricular zone (SVZ). In humans and other gyrencephalic lineages, the SVZ is divided by a fibrous layer into an inner SVZ (iSVZ) and outer SVZ (oSVZ). The oRGCs have one long basal process connecting them to the pial surface, whereas the IPCs are multipolar and do not contact either cortical surface (Hansen et al., 2010). The vRGC population uses their processes to undergo migratory nuclear activity with apicobasal directionality in accordance with their stage in the cell cycle, a process termed INM (Ueno et al., 2006). For instance, the nuclei of vRGCs in the S phase will be located basally (Figure 2A). As the cell cycle progresses and the progenitor enters the G2 stage, the nucleus starts migrating toward the ventricular surface where mitosis takes place (Figures 2B,C). After mitotic division, the nucleus migrates in the basal direction as it progresses through the G1 phase (Lui et al., 2011; Spear and Erickson, 2012a,b; Doobin et al., 2016; Jiang and Nardelli, 2016).

The apical migratory activity of vRGCs is dictated by two different yet complementary mechanisms that become active at different stages of INM (Figure 2). The first mechanism is initiated during the early G2 phase, in which CDK1 phosphorylates the nucleoporin RanBP2 (Bertipaglia et al., 2018). This phosphorylation event allows RanBP2 to interact with protein bicaudal D homolog 2 (BicD2) (Figure 2B,B’), which acts as an adapter protein that links the dynein motor complex to various cargos. Interaction between phosphorylated RanBP2 and BicD2 recruits Lis1 and dynein, forming the RanBP2/BicD2/LIS1/dynein motor complex that initiates apical migration (Bertipaglia et al., 2018; Figure 2B’). The secondary mechanism, which is triggered during late G2, involves Nde1 participation in apical nuclear migration. At this point, CenP-F binds to Nde1 and the nucleoporin Nup133 at the nuclear envelope (Figure 2C,C’) (Bolhy et al., 2011). Binding of CenP-F, Nup133, and Nde1 will recruit dynein to form the Nde1/Lis1/dynein motor complex (Figure 2C’). Specifically, CenP-F acts as an anchor to the nucleus, via its interaction with Nup133, to pull this organelle toward the ventricular surface along the microtubules (Kim et al., 2011; Bertipaglia et al., 2018; Doobin and Vallee, 2018). In turn, these microtubules are anchored at the centrosome, which itself is anchored by the primary cilium (Kim et al., 2011; Spear and Erickson, 2012a,b). In sum, the first half of apical nuclear migration appears to occur independent of NDE1 activity, but the latter half requires it. In late G2, just before M-phase, high levels of NDE1 will cause the primary cilium to disassemble, releasing the centrosome from the apical surface (Kim et al., 2011). The released centrosome travels basally until it reaches the nucleus, where nuclear envelope breakdown initiates followed by subsequent mitosis (Spear and Erickson, 2012a,b).

It is thought that disrupting the migration dynamics of INM can result in reduced progenitor pool size (Murciano et al., 2002). However, the exact mechanism by which this occurs remains an open question. Although the idea that progenitors undergo INM to reach the apically located centrosome is possible, recent reports have suggested that mitotic entry is not restricted to the ventricular surface (Strzyz et al., 2015). Ectopic mitosis has been observed when INM is impaired, which suggests that the apically located centrosome migrates toward the location of the nucleus (Spear and Erickson, 2012b). However, ectopic mitoses are associated with shifts toward differentiative divisions, and this change in progenitor behavior has been connected to the extracellular signal gradient progenitor cells that are normally exposed to while undergoing INM (Bene et al., 2008). For instance, genetic ablation of *Nde1* in mice caused ectopic mitoses, increased cell cycle exit, and increased production of early born cortical neurons, including both Cajal–Retzius cells and neurons of the cortical plate, at the expense of cortical progenitor cells (Feng and Walsh, 2004; Pawlisz et al., 2008). Therefore, *Nde1* deletion is linked to both ectopic mitoses and increased neurogenic drive in the early developing mouse cortex. It is intriguing to note that although most Cajal–Retzius cells are produced from extra-cortical sites, Pawlisz et al. (2008) traced the increased Cajal–Retzius cell production to the *Nde1*-null ventricular zone (Hevner et al., 2003; Bielle et al., 2005; Gil et al., 2014). Currently, little is known about whether *Nde1* may regulate Cajal–Retzius fate specification in cortical progenitors or the molecular mechanisms that might underlie such a role.

The depletion of the RGC progenitor pool is also likely to impair neuronal migration as a secondary effect. During cortical development, progenitor populations produce neurons that migrate basally to form the six neuronal layers of the cortex (Nadarajah, 2003). Newborn neurons require scaffolding for their migration from the VZ and SVZ to their respective neuronal layer. This scaffolding is formed by the basal processes of vRGCs and oRGCs, and the migratory process is known as the radial pathway (Viot et al., 2004). Therefore, the loss of these progenitor populations would reduce the quantity of these basal fibers (Jiang and Nardelli, 2016). As a result, this would also limit the migratory capacity of newborn neurons, producing a delaminated cortex. Accordingly, clinical evidence has shown that patients with *NDE1* pathogenic variants do exhibit disorganized cortical layers (Bakircioglu et al., 2011).

## A Potential Role of *Nde1* in Progenitor Cell Behavior

In mice, depleting *Nde1* results in microcephaly, albeit to a relatively lesser degree than in humans (Feng and Walsh, 2004; Pawlisz et al., 2008). Studies performed on *Nde1*-null mice have shown that the reduction in brain size is not due to cell death but rather the failure of progenitor cells to replenish the progenitor pool, causing its rapid depletion (Feng and Walsh, 2004). A likely explanation for this phenomenon is that loss of *Nde1* shifts RGCs from proliferative to neurogenic divisions, which would explain the overproduction of neurons born in the early stages of corticogenesis observed in *Nde1*-null mice (Pawlisz et al., 2008). The specific contribution of *Nde1* function to the regulation of progenitor cell behavior has not yet been definitively established. Although not definitively tested, possible mechanisms to explain how *Nde1* could affect proliferative vs. neurogenic division decisions in RGCs include: (i) mitotic defects and delays, (ii) prolonged G1-S transition, and (iii) altered centrosomal orientation during mitosis.

It has been shown that *Nde1* depletion causes mitotic defects in progenitor cells and affects mitotic spindle assembly and proper chromosome segregation (Feng and Walsh, 2004). Indeed, loss of *Nde1* in the developing mouse cortex results in mitotic delays, alongside an increase in differentiative divisions of vRGCs at the expense of proliferation (Feng and Walsh, 2004; Pawlisz et al., 2008). In support of this notion, experiments utilizing mitotic inhibitors in cortical progenitor cells have shown that prolonging the duration of mitosis drives progenitors to undergo differentiative divisions instead of proliferative ones (Pilaz et al., 2016). Similarly, it is possible that the increase in early neurogenesis observed in the cortex of *Nde1*-null mice is the result of changes in mitotic behaviors such as mitotic delays that drive differentiation (Feng and Walsh, 2004; Pawlisz et al., 2008; Pilaz et al., 2016).

In addition to the temporal regulation of mitosis, prolongation of other cell cycle phases can influence progenitor fate decisions. For example, changing the length of the G1 phase triggers changes in progenitor division type, with prolongation linked to a shift to differentiative divisions and shortening linked to proliferative divisions (Salomoni and Calegari, 2010). Therefore, the timing of the G1-S phase transition can powerfully affect progenitor cell behavior. The transition from G1 to S phase is controlled in part by the primary cilium (Kim et al., 2011). Throughout cell cycle progression, the non-motile primary cilium undergoes structural assembly and disassembly (Pan and Snell, 2007). For progenitors to undergo proper G1-S transition, the primary cilium must be resorbed (Li et al., 2011). Thus, if deciliation does not occur, delayed exit from G1 in progenitor cells could result in a bias toward differentiative divisions and subsequent depletion of the progenitor pool. Studies have shown that *Nde1* is a negative regulator of primary cilium dynamics and that loss of *Nde1* prevents proper deciliation (Kim et al., 2011; Doobin et al., 2016; Gabriel et al., 2016). This notion is consistent with the expression pattern of *NDE1*, where expression gradually increases through G2, peaking at and dropping after mitosis. Although not definitively tested, loss of Nde1 may cause lengthening of the G1 phase, which subsequently causes progenitors to shift toward differentiative divisions (Kim et al., 2011).

It has also been proposed that *Nde1* influences progenitor cell fate divisions by controlling mitotic spindle dynamics, particularly the orientation of the mitotic cleavage plane (Feng and Walsh, 2004). Normally, the majority of progenitor cells in anaphase exhibit cleavage planes perpendicular to the ventricular surface (symmetric divisions), which produce two daughter cells of the same type and, in RGCs, allows for self-amplification (Jiang and Nardelli, 2016). In contrast, cleavage planes parallel to the ventricular surface (asymmetric divisions) are primarily associated with differentiative divisions utilized by RGCs (Huttner and Brand, 1997; Feng and Walsh, 2004; Pawlisz et al., 2008). Altering the mitotic cleavage plane results in the asymmetric inheritance of intracellular cell fate determinants, which are localized in the apical surface of progenitor cells (Chenn and Mcconnell, 1995; Ahringer, 2003; Pawlisz et al., 2008). Asymmetric inheritance of intracellular components is known to determine progenitor cell fate (Lui et al., 2011), and a shift toward asymmetric, differentiative division would deplete the progenitor pool (Feng and Walsh, 2004). *Nde1* loss-of-function models exhibit misaligned mitotic chromosomes and changes in mitotic plane orientation (Feng and Walsh, 2004; Pawlisz et al., 2008). In particular, *Nde1*^–/–^ progenitors exhibit a shift to cleavage planes parallel to the ventricular surface (Feng and Walsh, 2004; Pawlisz et al., 2008). Based on our current understanding, it seems likely that at the organismal level, *Nde1* plays an indispensable role in establishing the progenitor pool by maintaining by organizing the mitotic spindle, and by extent, the orientation of the mitotic cleavage plane, in a manner that favors symmetric proliferative divisions (Lamonica et al., 2013). Mitotic spindle disorientation observed when *Nde1* is lost may stem from disruption of microtubule focusing onto the kinetochore, due to loss of the connection linking CENP-F to the LIS1/dynein motor complex (Feng and Walsh, 2004; Vergnolle and Taylor, 2007; Monda and Cheeseman, 2018).

## In Humans, Loss of *Nde1* Function Causes Severe Microcephaly

**FIGURE 3:**
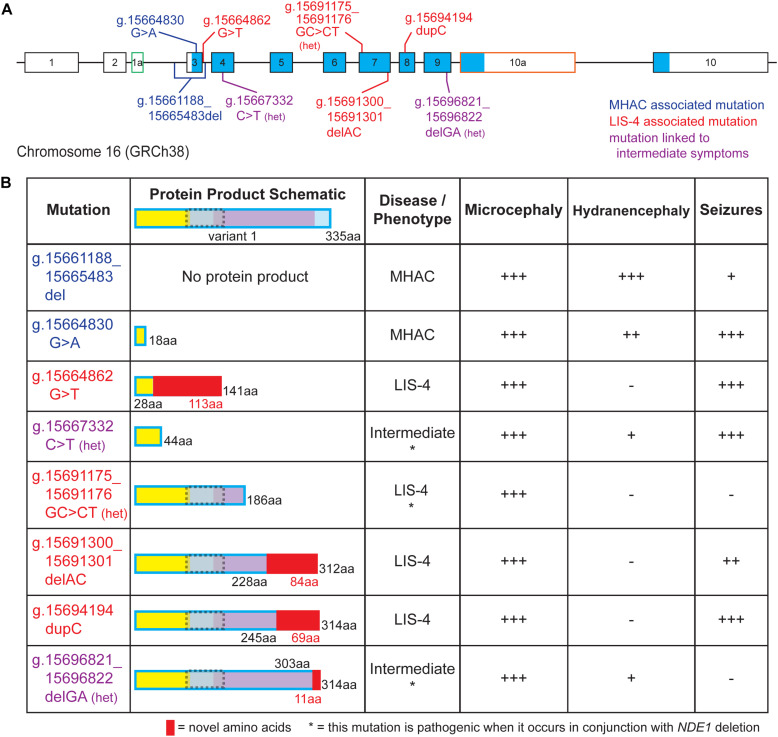
Pathological mutations in *NDE1* are linked to one of three clinical profile categories. **(A)** Schematic of human *NDE1*, including exons from isoforms 1, 2, and X2. Exons outlined in black are found in the canonical isoform (isoform 1), whereas alternative exons have either green or orange outlines. White fill indicates an untranslated region, whereas blue fill indicates the open reading frame. Mutations are color-coded by associated clinical profile type (blue = microhydranencephaly, or MHAC; red = lissencephaly-4, or LIS-4; and purple = intermediate). **(B)** Table of *NDE1* mutations from **(A)**, schematics of their expected protein product, their associated disease type, and presence and severity of microcephaly, hydranencephaly, and seizures in patients carrying the mutation. Protein schematics are color-coded by self-associated domain (yellow), LIS1-interacting domain (gray with dotted gray outline), NUDE_C domain (purple), sequence unassigned to a functional domain (blue), or novel sequence in the mutant protein (red). “Het” or * notes compound heterozygous mutations that are pathogenic when occurring alongside microdeletion of the *NDE1* locus. All other mutations are pathogenic when homozygous. Symptom severity is noted as follows: –, not reported; +, mild; ++, moderate; +++, severe.

Pathogenic variants of *NDE1* have been linked to congenital microcephaly in humans (Alkuraya et al., 2011; Bakircioglu et al., 2011; Guven et al., 2012; Tan et al., 2017; Abdel-Hamid et al., 2019). Although *Nde1*^–/–^ mice also exhibit decreased cortical size (Feng and Walsh, 2004; Pawlisz et al., 2008), the microcephaly observed in patients is much more severe, which suggests *NDE1* may play a more crucial role in the cortical development of humans than it does in mice (Mosca et al., 2017). Currently, eight pathogenic variants of *NDE1* have been reported in clinical studies. These mutations vary in their clinical manifestation, where microcephaly presents with lissencephaly or hydranencephaly, in which cerebral structures are replaced with cerebrospinal fluid (Barkovich and Raybaud, 2012). Additional symptomatology observed in these patients includes intellectual disability and ventriculomegaly, and many of them also exhibit seizures (Figure 3). Due to the multiple clinical manifestations caused by *NDE1* mutations, clinical profiles of patients have been grouped into three categories: (1) microhydranencephaly (MHAC), characterized by microcephaly and hydranencephaly; (2) lissencephaly-4, which is characterized by congenital microcephaly with lissencephaly (microlissencephaly); and (3) “Intermediate,” in which characteristic symptoms of both lissencephaly-4 and MHAC are observed (Paciorkowski et al., 2013).

Two distinct mutations in *NDE1* have been associated with MHAC (Figure 3B; Guven et al., 2012; Abdel-Hamid et al., 2019). Three related siblings with homozygous g.15661188_15665483del *NDE1* mutations were identified by Guven et al. (2012) and two siblings with a homozygous nonsense variant (g.15664830 G > A) were identified by Abdel-Hamid et al. (2019); both mutations were reported to result in MHAC (Figures 3A,B). The clinical profile of these patients is characterized by congenital microcephaly, bilateral hydranencephaly, ventriculomegaly, severe cortical hypoplasia, abnormal gyrification, intellectual disability, seizures, and brainstem and cerebellar hypoplasia (Figure 3B; Guven et al., 2012; Abdel-Hamid et al., 2019).

Four pathogenic variants of *NDE1* linked to lissencephaly-4 have been described (Figures 3A,B; Alkuraya et al., 2011; Bakircioglu et al., 2011; Tan et al., 2017). Three of these are frameshift mutations (g.15664862G > T, g.15691300_15691301delAC, and g.15694194dupC). For three of these mutations, autosomal recessive inheritance has been linked to primary microcephaly with disordered cortical lamination (Figures 3A,B; Alkuraya et al., 2011; Bakircioglu et al., 2011). Meanwhile, the remaining mutation associated with lissencephaly-4 (g.15691175_15691176GC > CT) stems from compound heterozygosity where one allele carries the mutation and the other allele is absent due to a microdeletion spanning the *NDE1* locus (Figures 3A,B; Tan et al., 2017). Patients with lissencephaly-4 exhibit congenital microcephaly, simplified gyrification, ventriculomegaly, agenesis of the corpus callosum, and intellectual disability. Many patients also exhibited seizures and hypoplasia of the cerebellum (Figures 3A,B; Alkuraya et al., 2011; Bakircioglu et al., 2011; Tan et al., 2017).

Finally, Paciorkowski et al. (2013) described two separate heterozygous *NDE1* mutations, a nonsense mutation (g.15667332C > T) and a frameshift mutation (g.15696821_15696822delGA), in unrelated patients, born from non-consanguineous phenotypically normal parents (Figure 3A). These patients had a chromosome 16 microdeletion spanning *NDE1* on one allele and one of these two mutations on the remaining allele; Paciorkowski et al. (2013) described the clinical profile of these patients as an intermediate between the lissencephaly-4 and MHAC phenotypes previously reported (Figure 3B; Alkuraya et al., 2011; Bakircioglu et al., 2011; Guven et al., 2012; Tan et al., 2017; Abdel-Hamid et al., 2019). These patients displayed congenital microcephaly, ventriculomegaly, simplified gyral patterns, cerebrospinal fluid-filled cavities, agenesis of the corpus callosum, and prominent scalp rugae (Figure 3B). Specifically, one patient displayed increased extra-axial space at the intrahemispheric fissure, whereas the other patient had a large interhemispheric cavity. One of them developed seizures, whereas the other only had one reported seizure episode (Paciorkowski et al., 2013).

Symptoms linked to *NDE1* mutations mirror some aspects of the clinical profile of patients with *LIS1* mutations. Whereas *LIS1* mutations generally manifest as classical lissencephaly (lissencephaly-1), characterized by disorganized lamination of the cortex without microcephaly, *NDE1* encephalopathy presents itself as extreme brain atrophy that includes microcephaly and lissencephaly (lissencephaly-4) (Saillour et al., 2009). Lissencephaly-1 is believed to be caused by disrupting the migration of neurons along the RGC apical fiber, also known as the radial pathway, during gestation (Saillour et al., 2009). Because *NDE1* mutations are expected to decrease the quantity of RGC fibers, the radial pathway for migrating neurons is also affected (Viot et al., 2004). That *NDE1* mutations not only mirror symptoms linked to *LIS1* mutations but also exhibit more severe symptoms, suggesting that these proteins, despite participating in the same functional pathway, differ in their contribution to cortical development.

## Molecular Differences in *Nde1* Pathogenic Variants

As the documented cases of *NDE1* mediated microcephaly increase, so does the need to understand the basic biology of these pathogenic variants. These variants are expected to differ in their degree of truncation, particularly in the C-terminal. One possibility is that the expected clinical profile associated with a given pathogenic variant of *NDE1* is contingent on which functional domains are compromised. Additionally, pathogenic variants that maintain most of their functional domains intact may reflect problems with the folding dynamics of the mutant protein (Soares et al., 2012). Although these ideas will remain speculative without the experimental evidence to back them, evaluating the protein product of these mutations and how they manifest clinically may reveal patterns worth studying in the future.

Due to the more severe clinical manifestations, much attention has been placed on mutations causing the MHAC phenotype. Guven et al. (2012) report an *NDE1* mutation (g.15696821_15696822delGA) that results in the deletion of the entire initial coding exon (Figures 3A,B; Guven et al., 2012). It is predicted that the loss of this exon results in a null allele incapable of producing a protein product. Similarly, the homozygous nonsense variant g.15664830G > A introduces a premature stop codon in the first protein-coding exon (exon 3) of the *NDE1* transcript (Figures 3A,B). This would produce a protein of only 18 native amino acid residues that lacks the LIS1 binding and NUDE-C domains and maintains only a minimal portion of the self-association domain (Figure 3B). In summary, *NDE1* variants associated with MHAC seem to be linked as an extreme case of loss-of-function, where *NDE1* protein is either completely absent or the protein product is likely too small to carry out any function.

Lissencephaly-4 is the most common clinical manifestation of *NDE1* mutations. Of the four mutations linked to this disease, three are predicted to disrupt the NUDE_C domain (g.15691175_15691176GC > CT, g.15691300_15691301delAC, and g.15694194 dupC), whereas the fourth mutation (g.15664862G > T) is expected to truncate at the N-terminal self-association domain (Figure 3B). The truncated NUDE_C domain encoded by these three variants is expected to disrupt interactions with CENP-F, dynein, and CDK1 (Hirohashi et al., 2006; Vergnolle and Taylor, 2007; Alkuraya et al., 2011; Bakircioglu et al., 2011; Figures 1B,C, 3B). Specifically, the frameshift mutation g.15691300_15691301delAC is found in exon 7; this mutation is expected to produce a protein that diverges from the canonical sequence at amino acid 228 and adds 83 non-native amino acid residues (Alkuraya et al., 2011; Bakircioglu et al., 2011; Figures 3A,B). Similarly, the g.15694194dupC mutation, which occurs in exon 8, diverges from the canonical sequence at amino acid 245 and adds 68 non-native amino acid residues (Figures 3A,B). Alkuraya et al. (2011) did show that for mutations g.15691300_15691301delAC and g.15694194 dupC, the *NDE1*-dynein interaction is lost, whereas the NDE1-LIS1 interaction is preserved (Alkuraya et al., 2011). Meanwhile, the mutation g.15691175_15691176GC > CT is predicted to result in a missense p.K185N mutation followed by a premature stop codon (p.Q186X), which are predicted to produce a truncated protein (Tan et al., 2017; Figures 3A,B). Notably, the pathogenicity of this heterozygous mutation has only been observed alongside the deletion of the other *NDE1* allele (Figure 3B). The final mutation linked to lissencephaly-4 is the g.15664862 G > T mutation, which affects the donor splice site of intron 3, located between exons 3 and 4 (Figure 3A). This splice site mutation is expected to result in a frameshift affecting amino acid 29 that adds 113 non-native amino acids to the NDE1 protein (Bakircioglu et al., 2011; Figure 3B). Given that only the first 28 amino acids on the N-terminus are native, the resulting protein lacks both LIS1 and NUDE_C domains and has profound truncation in its self-association domain. It is evident that variants associated with lissencephaly-4 exhibit discrepancies among the compromised domains. We note the addition of non-native amino acid residues and/or disrupting the NUDE-C domain as potential determinants of this clinical profile. Nonetheless, future studies will be required to interrogate potential mechanisms by which pathogenic variants result in the same clinical outcome despite being compromised at different locations.

Mutations responsible for the “intermediate” disease are located in substantially different regions of *NDE1* (Paciorkowski et al., 2013). The nonsense mutation g.15667332 C > T results in truncation at amino acid residue 44, disrupting the self-association domain (Figure 3B). This protein variant lacks the LIS1-binding region and the NUDE-C domain. Meanwhile, the frameshift mutation g.15696821_15696822delGA located in exon 8 is expected to truncate the NUDE_C domain, with divergence at amino acid 303 and the addition of 11 non-native amino acids (Paciorkowski et al., 2013; Figure 3B). The protein product of this mutation might allow dimerization and LIS1 binding, but the functionality of its NUDE-C domain is questionable (Figures 1B,C, 2B). The latter two mutations result from one of the *NDE1* alleles being deleted while the remaining allele carries the mutation. Due to the heterozygosity of the mutant allele, the expression of the mutated protein in these patients is likely decreased relative to patients with homozygous mutations. Thus, the compounding effects of compromised function and decreased expression could be responsible for this clinical profile. It is worth mentioning that although the g.15691175_15691176GC > CT mutation associated with lissencephaly-4 also occurs in the absence of one *NDE1* allele, the degree of truncation is not as severe as g.15667332 C > T and does not have any non-native residues such as g.15696821_15696822delGA.

In all, although the MHAC phenotype appears to be linked to severe loss-of-function of *NDE1*, the more moderate loss-of-function resulting from truncation of the NUDE-C domain and the addition of novel amino acid residues seems to underlie lissencephaly-4 (Figure 3B). Notably, the additional 113 non-native amino acids in the expected protein product of the g.15664862G > T mutation, also associated with lissencephaly-4, could potentially impart a toxic function to the protein (Figure 3B). On the other hand, an association between the “intermediate” mutations and the observed symptom severity is not clear-cut. For instance, despite the product of the g.15667332C > T mutation only maintaining 44 amino acids of the self-association domain, it is possible this truncated protein is still able to dimerize with and sequester NDEL1, potentially disrupting its cellular functions as well (Figures 1B,C, 3B). On the other hand, the protein product of the g.15696821_15696822delGA mutation also manifests as the “intermediate” phenotype, yet it maintains almost all of its functional domains intact except the final 32 amino acids at its C-terminal (Figure 3B). Given that most of the protein is kept intact in this case, it is possible that the short addition of non-native amino acids at the C-terminal leads to a loss-of-function in the NUDE-C domain, which could interrupt the folding dynamics and/or abolish interaction with the 26S proteasome. Why these variants manifest in a similar “intermediate” clinical profile despite the pronounced difference in protein product is currently unknown. However, the symptoms associated with the compound heterozygous mutations deemed “intermediate” vary based on the mutation. For instance, g.15667332C > T is linked to seizures, which was not observed in the patient with the g.15696821_15696822delGA mutation, which would produce a mostly intact *NDE1* protein (Figure 3B). Future research is required to better elucidate the spectrum of clinical profiles associated with *NDE1* mutations and to definitively identify any existing relationships between a clinical profile and protein product.

## Phylogenetic Relationship Between *Nde1* and High-Order Gyrification

*Nde1* contributes significantly to neurodevelopment in mammalian species. Multiple studies suggest that the functions of *NDE1* are conserved throughout evolution. For example, the role of *NDE1* in both nuclear migration and mitotic spindle regulation is well-conserved across eukaryotes (Morris et al., 1995; Alkuraya et al., 2011; Xiang, 2018). Meanwhile, evidence indicates that the kinetochore-binding function of *NDE1* is conserved from Ecdysozoa to Primates (Wainman et al., 2009; Simões et al., 2017). Despite the conservation of *NDE1* functions, its role at the organismal level differs across evolution. Therefore, it is possible that universal functions of *NDE1* across evolution are mediated by conserved interactions with binding partners. In this case, one would expect the domains responsible for these interactions to be strongly conserved across evolution.

**FIGURE 4:**
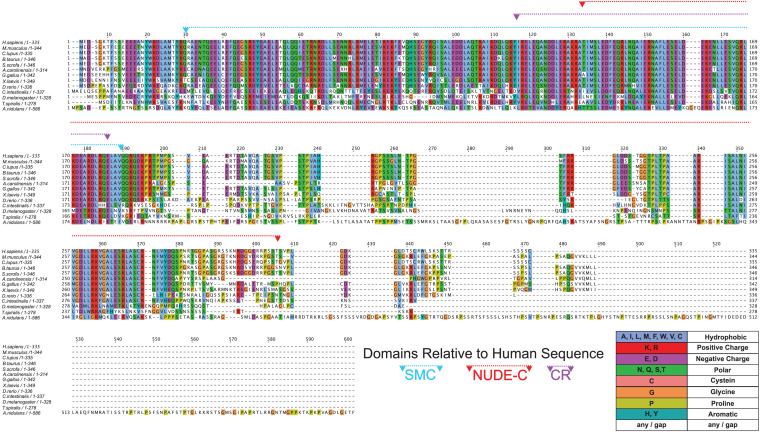
Conservation of NDE1 across Opisthokonta. Protein alignment of NDE1 orthologs in fungal (*A. nidulans*), ecdysozoan (*T. spiralis*, *D. melanogaster*), ascidian (*C. intestinalis*), and vertebrate (*D. rerio, X. laevis, A. carolinensis, G. gallus, B. taurus, S. scrofa, C. l. familiaris, M. musculus*, and *H. sapiens*) species. Sequences are color-coded by property using ClustalX coloring in Jalview. Conserved domain superfamilies are represented in relation to the human sequence. CR, conserved region.

To test this possibility, we first used the NCBI Conserved Domain web tool^1^ to determine which domains are present in human NDE1. This analysis revealed the presence of NUDE-C and structural maintenance of chromosome (SMC) superfamily domains in the human NDE1 sequence. The NUDE-C domain is found at the C-terminal region of the NUDE protein family, and it has been reported to play multiple cellular roles, including nuclear migration and mitosis (Figure 1B). On the other hand, the SMC domain superfamily has been attributed to chromosome segregation functions, cell cycle control, and chromosome partitioning. In the human NDE1 protein, the SMC superfamily domain spans amino acids Q27 to Q186, encompassing most of the self-association domain, the entire LIS1-binding region, and a small section of the NUDE-C domain (Figures 1B, 4).

**FIGURE 5:**
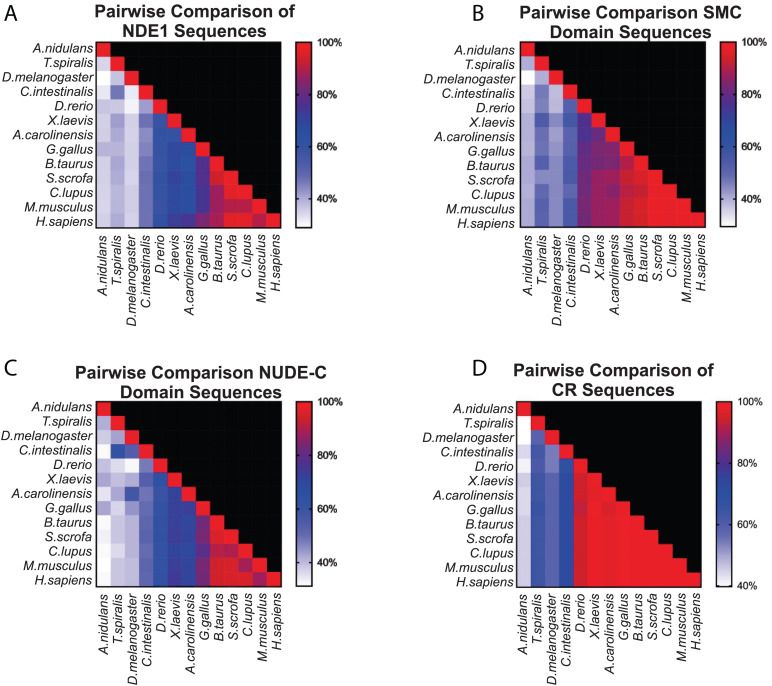
Pairwise comparison of SMC and NUDE_C domain sequences in vertebrates. **(A–D)** Matrices showing the percentage homology in pairwise species comparisons of **(A)** the entire protein sequence of the respective NDE1 ortholog; **(B)** the SMC domain sequences; **(C)** the NUDE_C domain sequences; and **(D)** the stretch of sequence with high conservation observed in Figure 4, spanning the end of the SMC domain to the beginning of the NUDE_C domain (K106 to K185 in the human NDE1 sequence). Color corresponds to the degree of sequence homology. CR, conserved region.

Once we established the conserved domains for human NDE1, we expanded the analysis to species spanning Opisthokonta, focusing primarily on metazoans. Utilizing ENSEMBL and NCBI, we examined NDE1 orthologs from the following species: the house mouse (*Mus musculus*), pig (*Sus scrofa*), domesticated dog (*Canis familiaris*), chicken (*Gallus gallus*), green anole (*Anolis carolinensis*), African clawed frog (*Xenopus laevis*), zebrafish (*Danio rerio*), the tunicate *Ciona intestinalis*, common fruit fly (*Drosophila melanogaster*), the nematode *Trichinella spiralis*, and the fungus *A. nidulans*. Conserved domains of orthologous NDE1 sequences were identified using the same means used for the human sequence. Sequence alignments were performed for the whole sequence of these orthologs using NCBI protein BLAST. Similar alignments were also performed for the NUDE-C and SMC domains individually. Percent identity scores for these alignments were plotted to an identity matrix (Figure 5). Pairwise sequence comparison across the orthologs revealed that mammalian species shared the highest homology in the overall NDE1 sequence (Figure 5A). We noted that homology across primates, carnivorans, and ungulates is higher than it is between primates and rodents, despite these species being evolutionarily closer (Figure 5A). As would be expected if binding partners/functions are evolutionarily retained, we found that the domains of NDE1 are more conserved than the overall protein. In particular, vertebrate species have a greater degree of conservation in the SMC domain (Figure 5B). Meanwhile, conservation of the NUDE-C domain is highest in mammalian lineages and mirrors more closely the overall protein conservation (Figures 5A,C). The SMC and NUDE-C domain superfamilies share a 52-residue overlap (S134–Q186, in the human sequence; Figure 4). Notably, the alignment of orthologous sequences using Clustal Omega revealed that the highest conserved region (CR) among Opisthokonta spans from K106 to K185 in the respective human sequence, encompassing the overlapping region of the SMC and NUDE-C superfamily domains (Figures 4, 5D). This CR is located at the expected LIS1-binding domain of the human sequence (Figures 1B, 4). LIS1 is an ancestral binding partner of NDE1; therefore, this CR may contribute to the NDE1-LIS1 interaction across Opisthokonta. Shorter conserved sequences across Opisthokonta were also noted, spanning from Q32 to Q55 (Figure 4). This sequence falls within the self-association domain and is most likely involved in the dimerization of NDE1 and its interaction with the dynein intermediate chain (Żyłkiewicz et al., 2011; Figures 1B, 4). Aside from these regions, we found minimal conservation across Opisthokonta in the rest of the sequence (Figures 4, 5A). Due to its higher conservation, the SMC domain likely underlies the universal roles of NDE1 that are shared among eukaryotes (Figure 5B). This is opposed to the NUDE-C domain, which has been less conserved throughout evolution (Figure 5C).

Conservation of NDE1 is observed to be independent of evolutionary distance (i.e., orthologs of ungulates, primates, and carnivorans are more similar to each other than orthologs of primates and rodents are). This underscores the possibility that NDE1 divergence is coupled to functional changes in these lineages. A study by Mosca et al. (2017) reports species-specific isoforms of *NDE1* in humans and mice. In the human canonical *NDE1* transcript, the 3′ terminal exon (exon 10 in Figure 1A) overlaps with the neighboring *MYH11* gene between its exons 31 and 32 (Figure 1A). This overlap is absent in the mouse canonical isoform, which utilizes an alternate exon equivalent to exon 10a in Figure 1A (Mosca et al., 2017). Usage of this alternative exon could allow NDE1 to carry out novel functional interactions. Monda and Cheeseman (2018) highlight that the human-specific terminal exon allows for isoform-specific interaction between NDE1 and the 26S proteasome, which is not observed in isoforms lacking this terminal exon. Similarly, Mosca et al. (2017) suggested that these alternative terminal sequences may differentially influence transcript stability and thereby impact NDE1 protein production/levels. Given these potential functions, Monda and Cheeseman (2018) proposed the idea that species-specific alternative *Nde1* isoforms may contribute differently to the neurodevelopmental processes of humans and mice. In agreement with this, *Nde1* has been shown to play a more prominent role in the development of gyrencephalic brains than it does for lissencephalic ones (Feng and Walsh, 2004; Pawlisz et al., 2008; Alkuraya et al., 2011; Bakircioglu et al., 2011; Guven et al., 2012; Tan et al., 2017; Abdel-Hamid et al., 2019). Alternative exon usage may mirror patterns of cortical complexity in mammalian species. In agreement with the principle of parsimony, it is believed that gyrencephally is an ancestral trait to all mammals (Lewitus et al., 2014). The presence of gyrencephalic and lissencephalic species within closely related taxonomic groups highlights that lissencephaly is a reemerging trait of cortical evolution (Zilles et al., 2013). Mechanisms responsible for gyrification remain elusive; however, it has been suggested that RGCs are key in this process (Borrell and Götz, 2014). Specifically, the vRGC and oRGC populations establish the proliferative regions (SVZ and OSVZ), and these proliferative zones determine cortical excitatory neuron quantity. Because gyrification requires expansion of the cortical surface, increasing the RGC pool and/or prolonging the period of proliferative divisions are potential mechanisms by which this demand can be met. We know that *Nde1* is important for the mitotic progression and cellular behavior of vRGCs and NECs. Therefore, we hypothesized that *Nde1* isoforms containing exon 10 (Figure 1A) are preferentially used by species with gyrencephalic cortices.

**FIGURE 6:**
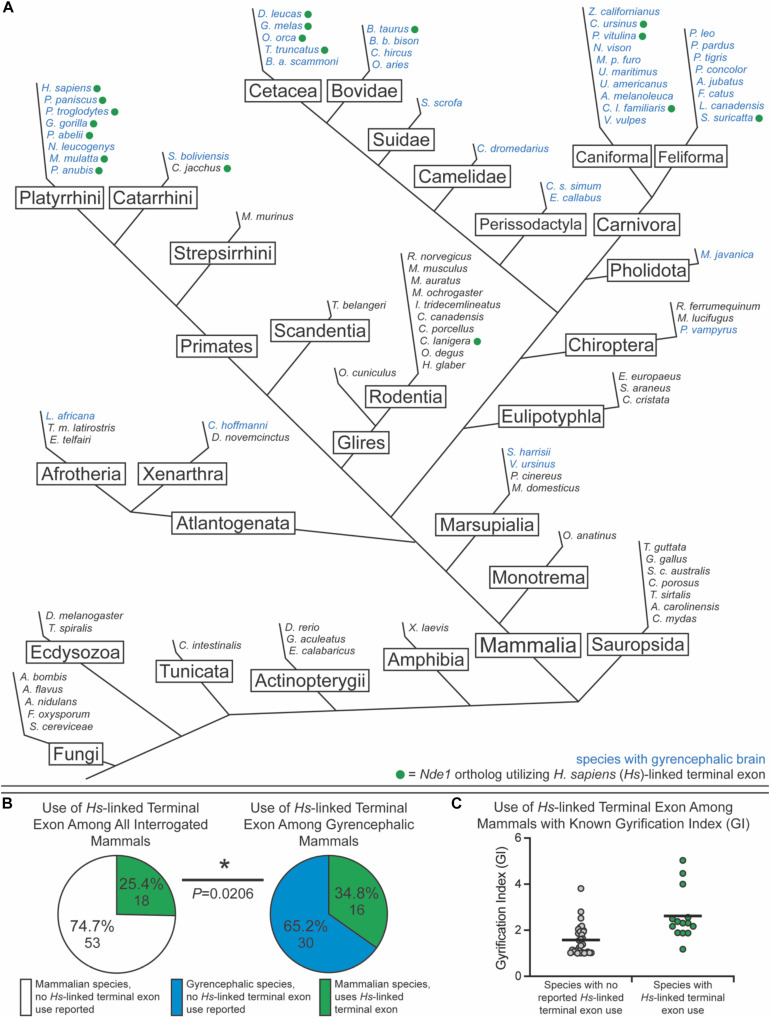
Usage of the human-linked alternate terminal exon tracks significantly with gyrencephaly. **(A)** Schematic phylogenetic tree of Opisthokonta, showing gyrencephalic species (blue text) and usage of the human (*Homo sapiens* [*Hs*])-linked alternate terminal exon (green circle). Branch lengths are not to scale. **(B)** Pie charts showing the percentage of species that utilize the human (*Hs*)-linked alternate *Nde1* terminal exon among all interrogated mammalian species (left) and among all gyrencephalic mammalian species (right). Fisher’s exact test determined statistical significance. *P < 0.05. **(C)** Distribution of gyrification index values across all sampled mammalian species with (green) and without (gray) reported human-linked *Nde1* terminal exon usage. Lines denote means.

To test this, we sought to classify species as either lissencephalic or gyrencephalic. For this, we curated published GI values for each genus; any species in a genus with a GI value above 1.2 was considered gyrencephalic (Figures 6A–C). When GI values were not available, brain image data from the Comparative Mammalian Brain Collection and published statements describing species as gyrencephalic or lissencephalic were used for this classification (Harper and Maser, 1976; Sullivan, 1982; Kawamoto et al., 1998; Phelps and Young, 2003; Xiao et al., 2006; Marino, 2007; Morawski et al., 2010; Lewitus et al., 2013; Zilles et al., 2013; Mota and Herculano-Houzel, 2015; Bhagwandin et al., 2017; Spocter et al., 2017; Raghanti et al., 2018; Ashwell and Gurovich, 2019; Marchand and Schwartz, 2019). The collection available at^2^ was used in our binary classification system. *Nde1* isoforms with transcript-level data were mined from NCBI and Ensembl. Based on the location of the isoform’s terminal exon, each isoform was binned as either overlapping with the *MYH11* ortholog (similar to the human-specific isoform previously described) or not (similar to the mouse-specific isoform) (Figures 6A–C). In total, we assessed the *Nde1* isoforms in 90 species in Opisthokonta, 71 of which were mammalian species. Of these mammalian species, 45 were classified as gyrencephalic, and 26 were binned as lissencephalic (Figure 6A).

**FIGURE 7:**
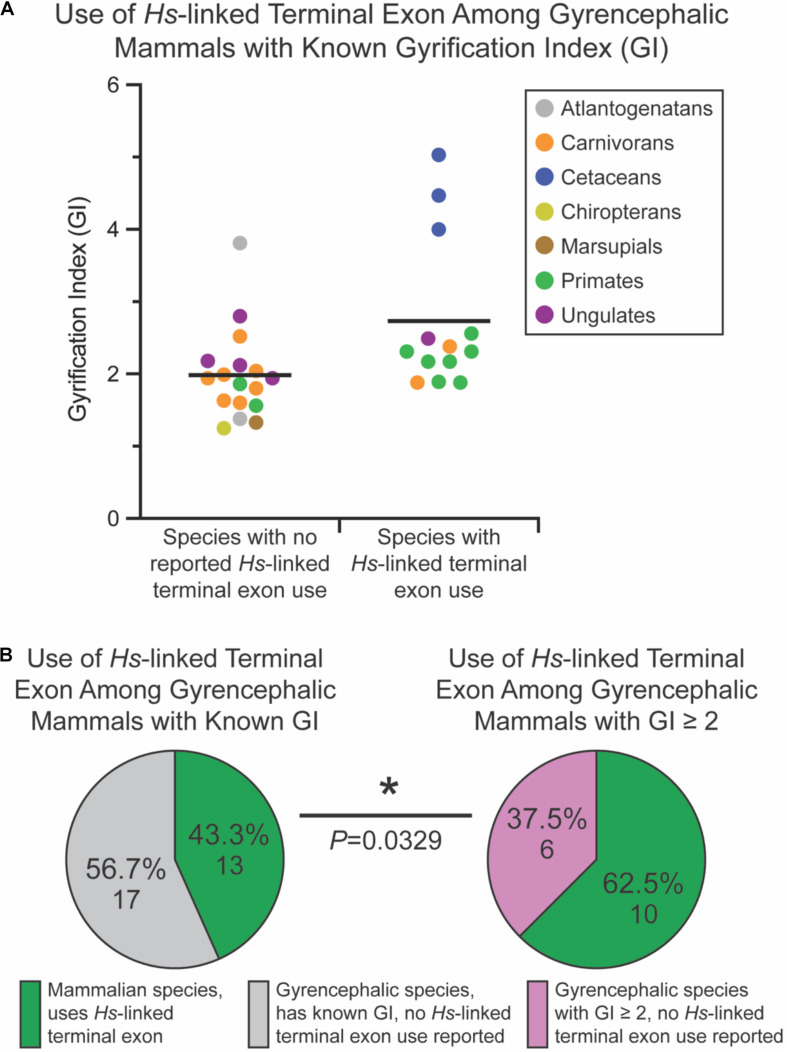
Usage of the human (*Homo sapiens [Hs]*)-linked terminal exon of *Nde1* is significantly higher among mammals with high degrees of gyrification. **(A)** Distribution of gyrification index (GI) values across sampled, gyrencephalic species with and without reported human-linked *Nde1* terminal exon usage. Each data point is color-coded by species clade. Lines indicate means. **(B)** Pie charts showing the percentage of species that utilize the human (*H. sapiens*)-linked alternate *Nde1* terminal exon among all interrogated gyrencephalic mammalian species with reported GI scores (left) and among gyrencephalic species with GI scores ≥ 2 (right). Fisher’s exact test determined statistical significance. *P < 0.05.

Of the non-mammalian species interrogated, none exhibited usage of the *Myh11*-overlapping human-linked terminal exon (equivalent to exon 10 in Figures 1A, 6A). The absence of this exon in non-mammalian species is in agreement with our hypothesis, given that gyrencephaly is a mammal-specific trait. Of the 71 mammalian species assessed, 25.4% utilized the human-linked terminal exon (Figures 6A,B). Cetacea, Carnivora, Euungulata, and Primates frequently exhibit the human-linked alternate terminal exon (Figures 6A, 7A). In contrast, this alternative terminal exon is not utilized in most rodents, except the chinchilla (Figure 6A). We then assessed the usage of the human-linked terminal exon in gyrencephalic mammals by Fisher’s exact test. Compared with all interrogated mammalian species, the gyrencephalic mammalian species were significantly more likely to utilize the human-linked terminal exon (34.8 vs. 24.5%; *P* = 0.0206; Figure 6B). Consistent with this trend, we observed that the average GI value of species utilizing the human-linked terminal exon was higher than the average GI of those that do not (2.62 vs. 1.58; Figure 6C). In addition to supporting the idea that *NDE1* isoforms may influence the development and evolution of gyrencephaly, these data also raise two possibilities: (1) the earliest mammals expressed the *Nde1* isoform with the *Myh11*-overlapping, human-linked terminal exon, and the mouse/lissencephaly-linked isoform evolved later to trigger the production of a lissencephalic brain; or (2) the gyrencephaly-linked isoform is essential for producing a highly gyrencephalic cortex, as opposed to simple gyrification, so they are found in lineages characterized by high gyrencephaly. In other words, the prevalence of the *Myh11*-overlapping, human-linked terminal exon in gyrencephalic species could be due to the conservation of the ancestral isoform, or it could be an emerging trait of species with high degrees of gyrification. To address this, we sought to classify the interrogated gyrencephalic mammals based on the degree of cortical gyrification. For this, we utilized reported GI scores, which were available for 30 of the 45 gyrencephalic mammalian species. Notably, even among gyrencephalic mammals, we still observed a higher average GI value among species utilizing the human-specific terminal exon, compared with gyrencephalic species without reported use of this exon (2.73 vs. 1.99; Figure 7A). To assess this relationship further, we binned all 30 gyrencephalic mammalian species as having either low (1.2 < GI < 2.0; 14 species) or high (GI ≥ 2.0; 16 species) degrees of gyrification. Of the species with reported GI scores, 43.3% exhibited usage of the human-linked terminal exon, and 53.3% met our threshold for high gyrification (Figures 7A,B). In contrast, usage of the human-linked terminal exon was observed in 62.5% of mammalian species with high degrees of gyrification, significantly higher than expected by Fisher’s exact test (*P* = 0.0329, Figure 7B). The high usage of the *Myh11*-overlapping, human-linked terminal exon in Cetacean and Primate lineages highlights its connections to species with a high GI (Figures 6A, 7A). Because an in-depth assessment of alternative terminal exon prevalence among species would require a high degree of sequencing work, it is important to note that our approach is susceptible to lineage-specific limitations in RNAseq expression data, as availability and quality of transcriptome data for rare species could drive artificial trends. With next-generation sequencing technology becoming faster and more accessible, future studies will be able to tease apart these relationships.

A mechanistic explanation for the functional roles of our gyrencephaly linked isoform remains to be definitively established. The *Myh11*-overlapping, human-linked exon is approximately 2.1 kbp in length, of which only 61 bp are translated. The rest of this sequence encodes 3′ untranslated region (UTR). Divergent 3′ UTR sequences found in these terminal exons may differentially regulate transcript integrity in a species-specific manner (Mignone and Pesole, 2011). Changes in the transcriptional regulation of *NDE1* likely disrupt its cellular functions (i.e., cell cycle regulation and mitotic progression) and, in turn, the proliferative program of progenitor populations. The amino acids encoded by the human-linked *Nde1* terminal exon do not correspond to the NUDE-C domain. Functional relevance for the amino acid sequence encoded by this gyrencephaly-linked exon has been proposed by Monda and Cheeseman (2018), who reported an interaction specifically between the gyrencephaly-linked isoform and the 26S proteasome but not the lissencephaly-linked isoform and this proteasome (Figures 1A,C). Monda and Cheeseman (2018) showed that removing the final 15 residues of the C terminus from the protein produced from the gyrencephaly-linked isoform was sufficient to disrupt this interaction with the 26S proteasome. Therefore, the interaction between NDE1 and the proteasome conceivably does not occur in species that lack the *Myh11*-overlapping, human-linked terminal exon. This species-specific terminal exon usage may inform *Nde1* functions related to gyrification, such as affecting the proliferative capacity of progenitor populations. Whether and how the NDE1-26S proteasome interaction contributes to cortical development remains to be definitively tested (Monda and Cheeseman, 2018).

## Conclusion and Future Directions

In this review, we have explored the roles of *Nde1* in progenitor cell behavior and mitosis, the genotype–phenotype relationships of *NDE1* associated developmental disorders, and most importantly, we identified relationships between the pattern of *NDE1* terminal exon usage and the development of highly gyrencephalic cortices. Although experimental findings in mouse models and cell culture have provided insight into the functional mechanism of *Nde1*, these studies do not recapitulate the pathophysiology of *NDE1* mutation-linked microcephaly in humans. This is due to the substantial architectural and cellular differences between the mouse and the human developing cortex, particularly their proliferative regions. For instance, as a lissencephalic species, mice lack a prominent oRGC population, which represents a predominant proliferative pool in humans and other gyrencephalic species such as the ferret (Feng and Walsh, 2004; Fietz et al., 2010; Hansen et al., 2010; Reillo et al., 2010; Wang et al., 2011; Betizeau et al., 2013; Lewitus et al., 2013). The expansion of the oRGC population has been linked to substantial increases in neuron production and likely underlies cortical expansion and gyrencephaly (Hansen et al., 2010; Tuoc et al., 2014; Llinares-Benadero and Borrell, 2019). Therefore, differences in the progenitor pool requirements for cortical development may account for the varying severity of *NDE1*-mediated microcephaly reported between humans and mice (Feng and Walsh, 2004; Alkuraya et al., 2011; Bakircioglu et al., 2011). To our knowledge, no experimental studies have been performed to evaluate how the expression of *NDE1* and its specific isoforms may affect the production of oRGCs and the establishment of the OSVZ. However, multiple experimental approaches have been developed in recent years that can be leveraged to address this question. For instance, the Kawasaki lab has published *in utero* electroporation strategies in ferrets, an exciting new model to study the genetic underpinnings of cortical folding (Kawasaki, 2018; Matsumoto et al., 2020). Another exciting new model system is the marmoset, a primate that has a lissencephalic cortex with a distinct OSVZ (Heide et al., 2020). The Brain/MINDS initiative has pioneered the generation of transgenic marmosets, and in collaboration with the Huttner group, they have shown that expansion of the oRGC pool in marmosets can drive the development of gyrencephaly (Okano et al., 2016; Heide et al., 2020). By harnessing these new models, future research will be able to reach more definitive conclusions regarding how *Nde1* shapes cortical development in gyrencephalic species.

Previous experimental findings and our findings have suggested that the usage of alternative *Nde1* terminal exons may inform cortical development differently in species of varying cortical complexity (Monda and Cheeseman, 2018). These data highlight gaps in our understanding of the functional mechanisms by which these isoforms function differently, such as the physiological relevance of the NDE1-26S proteasome interaction. It is also possible that evolutionary changes in *Nde1* primarily involve transcript expression, which itself could be affected by diverging 3′ UTR. For example, although it has been shown that the 3′ UTR can affect transcript integrity and turnover (Moor et al., 2005), the impact alternate *Nde1* 3′ UTR sequences have at the cellular level not been described. Another possibility worth looking into is whether *Nde1* isoforms co-evolved with specific transcriptional machinery to control terminal transcription stop site choice, and if this is the case, how this machinery would differentially influence aspects of trait evolution in these lineages. Addressing these unresolved questions will close important knowledge gaps and allow us to understand the role of *Nde1* evolution and its potential role as a contributor to gyrencephaly.

Although this review does not address the postnatal roles of *NDE1*, it should be mentioned that multiple reports have proposed a link between *NDE1* and psychiatric conditions through direct and indirect mechanisms (Bradshaw et al., 2009, 2019). Particularly, the interaction between disrupted in schizophrenia 1 (DISC1) and NDE1’s NUDE-C domain has been shown and has garnered attention, as it potentially relates *NDE1* to schizophrenia (Bradshaw et al., 2009, 2019; Thomson et al., 2012). Studies have shown that oligodendrocyte differentiation is impaired in schizophrenia (Davis and Haroutunian, 2003; Hattori et al., 2014; Mauney et al., 2015) and that DISC1 negatively regulates the differentiation of oligodendrocyte precursors (Porteous and Millar, 2009). Moreover, although *NDE1* promotes oligodendrocyte process formation (Shimizu et al., 2018), DISC1 has the opposite effect (Hattori et al., 2014). Although the mechanism by which NDE1-DISC1 interactions could contribute to schizophrenia remains unclear, it is conceivable that NDE1 contributes to the morphological differentiation of oligodendrocytes by sequestering DISC1. On a similar note, Bradshaw et al. (2019) reported a miRNA (mi-R484) located in the *NDE1* locus that is believed to alter psychiatric medications’ response by influencing their metabolism. The extent to which *NDE1* actively contributes to psychiatric disorders is still, for the most part, an unresolved question that will require *in vivo* studies to obtain a definitive answer.

Over the past decade, researchers have been developing organoid systems that allow modeling a multitude of tissue types (Simian and Bissell, 2016). Organoid systems can model the developing cortex in a manner that recapitulates anatomical and physiological integrity. As a result, cerebral organoids have been used to interrogate neurodevelopmental processes such as cortical folding and progenitor pool expansion and model disorders of these developmental processes (Hattori, 2013; Li et al., 2017; Borrell, 2018). For instance, cerebral organoids derived from patients with Miller–Dieker syndrome, which is characterized by severe lissencephaly, have successfully been used to model and study lissencephaly *in vitro* (Bershteyn et al., 2017). Similar model systems can be used to study the pathophysiology associated with *NDE1* mutations and may provide useful in elucidating the functional relevance of the *NDE1* alternative terminal exon (Kim et al., 2020). The latter would be facilitated by using a mutation identified by Monda and Cheeseman (2018) that abolishes the interaction between NDE1 and the 26S proteasome. An even more exciting possibility is emerging with the advent of multispecies organoids, which allow modeling of cortical development across evolution (Mora-Bermúdez et al., 2016; Pollen et al., 2018; Kanton et al., 2019; Muchnik et al., 2019; Benito-Kwiecinski et al., 2020; Eze et al., 2020) and may hold insight into the specific contributions of *NDE1* to cortical development. Although most multispecies organoid studies have sought to compare human and non-human primate cortical development, it is exciting to consider expanding this technology to other lineages with substantial rates of gyrification to study mechanistic overlap in the development and evolution of the gyrencephalic brain. In sum, although the present work suggests a phylogenetic relationship between *Nde1* isoforms and cortical development, additional research endeavors are required to interrogate isoform-specific functions that are beyond the previously described roles of *Nde1*.

## Data Availability Statement

Publicly available datasets were analyzed in this study. This data can be found here: https://www.ncbi.nlm.nih.gov/gene/54820/ortholog/?scope=7776 and https://uswest.ensembl. org/Homo_sapiens/Gene/Compara_Ortholog?db=core;g=ENSG 00000072864;r=16:15643267-15734691.

## Author Contributions

All authors contributed to the conception of the present manuscript. JS-P and MB reviewed the literature and wrote the draft of the manuscript. RK supervised the work and provided conceptual guidance.

## Conflict of Interest

The authors declare that the research was conducted in the absence of any commercial or financial relationships that could be construed as a potential conflict of interest.

## Footnotes

1 https://www.ncbi.nlm.nih.gov/Structure/cdd/wrpsb.cgi

2 http://neurosciencelibrary.org/Specimens/index.html

## References

[B1] Abdel-Hamid M. S. El-Dessouky S. H. Ateya M. I. Gaafar H. M. Abdel-Salam G. M. (2019). Phenotypic spectrum of NDE1-related disorders: from microlissencephaly to microhydranencephaly. *Am. J. Med. Genet. Part A* 179 494–497. 10.1002/ajmg.a.61035 30637988

[B2] Ahringer J. (2003). Control of cell polarity and mitotic spindle positioning in animal cells. *Curr. Opin. Cell Biol.* 15 73–81. 10.1016/s0955-0674(02)00018-2 12517707

[B3] Alkuraya F. S. Cai X. Emery C. Mochida G. H. Al-Dosari M. S. Felie J. M. (2011). Human mutations in NDE1 cause extreme microcephaly with lissencephaly. *Am. J. Hum. Genet.* 88:677 10.1016/j.ajhg.2011.04.020 PMC3146728 21529751

[B4] Ashwell K. W. Gurovich Y. (2019). Quantitative analysis of forebrain pallial morphology in monotremes and comparison with that in therians. *Zoology* 134 38–57. 10.1016/j.zool.2019.04.002 31146906

[B5] Bakircioglu M. Carvalho O. P. Khurshid M. Cox J. J. Tuysuz B. Barak T. (2011). The essential role of centrosomal NDE1 in human cerebral cortex neurogenesis. *Am. J. Hum. Genet.* 88 523–535. 10.1016/j.ajhg.2011.03.019 21529752 PMC3146716

[B6] Barkovich A. J. Raybaud C. (2012). “Congenital malformations of the brain and skull,” in *Pediatric Neuroimaging*, 5th Edn, eds Barkovich A. J. Raybaud C. (Philadelphia: Lippincott Williams &Wilkins), 367–568.

[B7] Bene F. D. Wehman A. M. Link B. A. Baier H. (2008). Regulation of neurogenesis by interkinetic nuclear migration through an apical-basal notch gradient. *Cell* 134 1055–1065. 10.1016/j.cell.2008.07.017 18805097 PMC2628487

[B8] Benito-Kwiecinski S. Giandomenico S. L. Sutcliffe M. Riis E. S. Freire-Pritchett P. Kelava I. (2020). An early cell shape transition drives evolutionary expansion of the human forebrain. *bioRxiv[Preprint].* 10.1101/2020.07.04.188078 PMC8054913 33765444

[B9] Bershteyn M. Nowakowski T. J. Pollen A. A. Lullo E. D. Nene A. Wynshaw-Boris A. (2017). Human iPSC-derived cerebral organoids model cellular features of lissencephaly and reveal prolonged mitosis of outer radial glia. *Cell Stem Cell* 20 435–449. 10.1016/j.stem.2016.12.007 28111201 PMC5667944

[B10] Bertipaglia C. Gonçalves J. C. Vallee R. B. (2018). Nuclear migration in mammalian brain development. *Semin. Cell Dev. Biol.* 82 57–66. 10.1016/j.semcdb.2017.11.033 29208348

[B11] Betizeau M. Cortay V. Patti D. Pfister S. Gautier E. Bellemin-Ménard A. (2013). Precursor diversity and complexity of lineage relationships in the outer subventricular zone of the primate. *Neuron* 80 442–457. 10.1016/j.neuron.2013.09.032 24139044

[B12] Bhagwandin A. Haagensen M. Manger P. R. (2017). The brain of the black (*Diceros bicornis*) and white (Ceratotherium simum) African rhinoceroses: morphology and volumetrics from magnetic resonance imaging. *Front. Neuroanat.* 11:74. 10.3389/fnana.2017.00074 28912691 PMC5583206

[B13] Bielle F. Griveau A. Narboux-Nême N. Vigneau S. Sigrist M. Arber S. (2005). Multiple origins of Cajal-Retzius cells at the borders of the developing pallium. *Nat. Neurosci.* 8 1002–1012. 10.1038/nn1511 16041369

[B14] Bodle J. C. Loboa E. G. (2016). Concise review: primary cilia: control centers for stem cell lineage specification and potential targets for cell-based therapies. *Stem Cells* 34 1445–1454. 10.1002/stem.2341 26866419

[B15] Bolhy S. Bouhlel I. Dultz E. Nayak T. Zuccolo M. Gatti X. (2011). A Nup133-dependent NPC-anchored network tethers centrosomes to the nuclear envelope in prophase. *J. Cell Biol.* 192 855–871. 10.1083/jcb.201007118 21383080 PMC3051818

[B16] Bomont P. Maddox P. Shah J. V. Desai A. B. Cleveland D. W. (2005). Unstable microtubule capture at kinetochores depleted of the centromere-associated protein CENP-F. *EMBO J.* 24 3927–3939. 10.1038/sj.emboj.7600848 16252009 PMC1283947

[B17] Borrell V. (2018). How Cells Fold the Cerebral Cortex. *J. Neurosci.* 38 776–783. 10.1523/jneurosci.1106-17.2017 29367288 PMC6596235

[B18] Borrell V. Götz M. (2014). Role of radial glial cells in cerebral cortex folding. *Curr. Opin. Neurobiol.* 27 39–46. 10.1016/j.conb.2014.02.007 24632307

[B19] Bradshaw N. J. Christie S. Soares D. C. Carlyle B. C. Porteous D. J. Millar J. K. (2009). NDE1 and NDEL1: multimerisation, alternate splicing and DISC1 interaction. *Neurosci. Lett.* 449 228–233. 10.1016/j.neulet.2008.10.095 19000741 PMC2631193

[B20] Bradshaw N. J. Hayashi M. A. F. (2016). NDE1 and NDEL1 from genes to (mal)functions: parallel but distinct roles impacting on neurodevelopmental disorders and psychiatric illness. *Cell. Mol. Life Sci.* 74 1191–1210. 10.1007/s00018-016-2395-7 27742926 PMC11107680

[B21] Bradshaw N. J. Hennah W. Soares D. C. (2013). NDE1 and NDEL1: twin neurodevelopmental proteins with similar ‘nature’ but different ‘nurture.’. *BioMolecular Concepts* 4 447–464. 10.1515/bmc-2013-0023 24093049 PMC3787581

[B22] Bradshaw N. J. Ukkola-Vuoti L. Pankakoski M. Zheutlin A. Ortega-Alonso A. Torniainen M. (2019). The Nde1 genomic locus can affect treatment of psychiatric illness through gene expression changes related to microrna-484. *Eur. Neuropsychopharmacol.* 29:170153 10.1016/j.euroneuro.2017.08.239 PMC5717342 29142105

[B23] Chenn A. Mcconnell S. K. (1995). Cleavage orientation and the asymmetric inheritance of notchl immunoreactivity in mammalian neurogenesis. *Cell* 82 631–641. 10.1016/0092-8674(95)90035-7 7664342

[B24] Cox J. Jackson A. P. Bond J. Woods C. G. (2006). What primary microcephaly can tell us about brain growth. *Trends Mol. Med.* 12 358–366. 10.1016/j.molmed.2006.06.006 16829198

[B25] Davis K. L. Haroutunian V. (2003). Global expression-profiling studies and oligodendrocyte dysfunction in schizophrenia and bipolar disorder. *Lancet* 362:758 10.1016/s0140-6736(03)14297-3 13678867

[B26] Dehay C. Kennedy H. (2007). Cell-cycle control and cortical development. *Nat. Rev. Neurosci.* 8 438–450. 10.1038/nrn2097 17514197

[B27] Derewenda U. Tarricone C. Choi W. C. Cooper D. R. Lukasik S. Perrina F. (2007). The structure of the coiled-coil domain of Ndel1 and the basis of its Interaction with Lis1, the Causal Protein of Miller-Dieker Lissencephaly. *Structure* 15 1467–1481. 10.1016/j.str.2007.09.015 17997972

[B28] Doobin D. J. Kemal S. Dantas T. J. Vallee R. B. (2016). Severe NDE1-mediated microcephaly results from neural progenitor cell cycle arrests at multiple specific stages. *Nat. Commun.* 7:12551. 10.1038/ncomms12551 27553190 PMC4999518

[B29] Doobin D. J. Vallee R. B. (2018). Cytoplasmic dynein and its regulators in neocortical development and disease. *Dyneins* 2018 262–285. 10.1016/b978-0-12-809470-9.00012-6

[B30] Efimov V. P. Morris N. R. (2000). The Lis1-Related Nudf Protein of Aspergillus nidulans Interacts with the Coiled-Coil Domain of the Nude/Ro11 Protein. *J. Cell Biol.* 150 681–688. 10.1083/jcb.150.3.681 10931877 PMC2175200

[B31] Eze U. C. Bhaduri A. Nowakowski T. J. Kriegstein A. R. (2020). Heterogeneity of Human Neuroepithelial Cells and Early Radial Glia. *bioRxiv[Preprint]*. 10.1101/2020.03.06.981423 PMC8012207 33723434

[B32] Feng Y. Walsh C. A. (2004). Mitotic Spindle Regulation by Nde1 Controls Cerebral Cortical Size. *Neuron* 44 279–293. 10.1016/j.neuron.2004.09.023 15473967

[B33] Fietz S. A. Kelava I. Vogt J. Wilsch-Bräuninger M. Stenzel D. Fish J. L. (2010). OSVZ progenitors of human and ferret neocortex are epithelial-like and expand by integrin signaling. *Nat. Neurosci.* 13 690–699. 10.1038/nn.2553 20436478

[B34] Finlay B. Darlington R. (1995). Linked regularities in the development and evolution of mammalian brains. *Science* 268 1578–1584. 10.1126/science.7777856 7777856

[B35] Fjell A. M. Westlye L. T. Amlien I. Tamnes C. K. Grydeland H. Engvig A. (2013). High-expanding cortical regions in human development and evolution are related to higher intellectual abilities. *Cereb. Cortex* 25 26–34. 10.1093/cercor/bht201 23960203

[B36] Gabriel E. Wason A. Ramani A. Gooi L. M. Keller P. Pozniakovsky A. (2016). CPAP promotes timely cilium disassembly to maintain neural progenitor pool. *EMBO J.* 35 803–819. 10.15252/embj.201593679 26929011 PMC4972140

[B37] Geschwind D. Rakic P. (2013). Cortical Evolution: judge the Brain by Its Cover. *Neuron* 80 633–647. 10.1016/j.neuron.2013.10.045 24183016 PMC3922239

[B38] Gil V. Nocentini S. Rio J. A. (2014). Historical first descriptions of Cajal–Retzius cells: from pioneer studies to current knowledge. *Front. Neuroanat.* 8:32. 10.3389/fnana.2014.00032 24904301 PMC4034043

[B39] Guven A. Gunduz A. Bozoglu T. M. Yalcinkaya C. Tolun A. (2012). Novel NDE1 homozygous mutation resulting in microhydranencephaly and not microlyssencephaly. *Neurogenetics* 13 189–194. 10.1007/s10048-012-0326-9 22526350

[B40] Hansen D. V. Lui J. H. Parker P. R. L. Kriegstein A. R. (2010). Neurogenic radial glia in the outer subventricular zone of human neocortex. *Nature* 464 554–561. 10.1038/nature08845 20154730

[B41] Harper J. W. Maser J. D. (1976). A macroscopic study of the brain of *Bison bison* bison, the American Plains Buffalo. *Anat. Rec.* 184 187–202. 10.1002/ar.1091840206 1247185

[B42] Hattori N. (2013). Cerebral organoids model human brain development and microcephaly. *Mov. Disord.* 29:185. 10.1002/mds.25740 24375826

[B43] Hattori T. Shimizu S. Koyama Y. Emoto H. Matsumoto Y. Kumamoto N. (2014). DISC1 (Disrupted-in-Schizophrenia-1) regulates differentiation of Oligodendrocytes. *PLoS One* 9:e0088506. 10.1371/journal.pone.0088506 24516667 PMC3917910

[B44] Hayashi M. A. F. Portaro F. C. V. Bastos M. F. Guerreiro J. R. Oliveira V. Gorrao S. S. (2005). Inhibition of NUDEL (nuclear distribution element-like)-oligopeptidase activity by disrupted-in-schizophrenia 1. *Proc. Natl. Acad. Sci. U.S.A.* 102 3828–3833. 10.1073/pnas.0500330102 15728732 PMC553309

[B45] Heide M. Haffner C. Murayama A. Kurotaki Y. Shinohara H. Okano H. (2020). Human-specific ARHGAP11B increases size and folding of primate neocortex in the fetal marmoset. *Science* 369 546–550. 10.1126/science.abb2401 32554627

[B46] Hevner R. F. Neogi T. Englund C. Daza R. A. Fink A. (2003). Cajal–Retzius cells in the mouse: transcription factors, neurotransmitters, and birthdays suggest a pallial origin. *Dev. Brain Res.* 141 39–53. 10.1016/s0165-3806(02)00641-7 12644247

[B47] Hirohashi Y. Wang Q. Liu Q. Li B. Du X. Zhang H. (2006). Centrosomal proteins Nde1 and Su48 form a complex regulated by phosphorylation. *Oncogene* 25 6048–6055. 10.1038/sj.onc.1209637 16682949

[B48] Huttner W. B. Brand M. (1997). Asymmetric division and polarity of neuroepithelial cells. *Curr. Opin. Neurobiol.* 7 29–39. 10.1016/s0959-4388(97)80117-1 9039800

[B49] Jiang X. Nardelli J. (2016). Cellular and molecular introduction to brain development. *Neurobiol. Dis.* 92 3–17. 10.1016/j.nbd.2015.07.007 26184894 PMC4720585

[B50] Kalebic N. Huttner W. B. (2020). Basal Progenitor Morphology and Neocortex Evolution. *Trends Neurosci.* 43 843–853. 10.1016/j.tins.2020.07.009 32828546

[B51] Kanton S. Boyle M. J. He Z. Santel M. Weigert A. Sanchís-Calleja F. (2019). Organoid single-cell genomic atlas uncovers human-specific features of brain development. *Nature* 574 418–422. 10.1038/s41586-019-1654-9 31619793

[B52] Kawamoto K. Kurahashi S. Hayashi T. (1998). Changes in the Gonadotropin-Releasing Hormone (GnRH) neuronal system during the annual reproductive cycle of the horseshoe bat, Rhinolophus ferrumequinum. *Zool. Sci.* 15 779–786. 10.2108/zsj.15.779

[B53] Kawasaki H. (2018). Molecular investigations of the development and diseases of cerebral cortex folding using gyrencephalic mammal ferrets. *Biol. Pharm. Bull.* 41 1324–1329. 10.1248/bpb.b18-00142 30175769

[B54] Kim J. Koo B. Knoblich J. A. (2020). Human organoids: model systems for human biology and medicine. *Nat. Rev. Mol. Cell Biol.* 21 571–584. 10.1038/s41580-020-0259-3 32636524 PMC7339799

[B55] Kim S. Zaghloul N. A. Bubenshchikova E. Oh E. C. Rankin S. Katsanis N. (2011). Nde1-mediated inhibition of ciliogenesis affects cell cycle re-entry. *Nat. Cell Biol.* 13 351–360. 10.1038/ncb2183 21394081 PMC3077088

[B56] Kiyomitsu T. Cheeseman I. M. (2012). Chromosome- and spindle-pole-derived signals generate an intrinsic code for spindle position and orientation. *Nat. Cell Biol.* 14 311–317. 10.1038/ncb2440 22327364 PMC3290711

[B57] Kriegstein A. Noctor S. Martínez-Cerdeño V. (2006). Patterns of neural stem and progenitor cell division may underlie evolutionary cortical expansion. *Nat. Rev. Neurosci.* 7 883–890. 10.1038/nrn2008 17033683

[B58] Lamonica B. E. Lui J. H. Hansen D. V. Kriegstein A. R. (2013). Mitotic spindle orientation predicts outer radial glial cell generation in human neocortex. *Nat. Commun.* 4:1665. 10.1038/ncomms2647 23575669 PMC3625970

[B59] Lewitus E. Kelava I. Huttner W. B. (2013). Conical expansion of the outer subventricular zone and the role of neocortical folding in evolution and development. *Front. Hum. Neurosci.* 7:424. 10.3389/fnhum.2013.00424 23914167 PMC3729979

[B60] Lewitus E. Kelava I. Kalinka A. T. Tomancak P. Huttner W. B. (2014). An adaptive threshold in mammalian neocortical evolution. *PLoS Biol.* 12:e1002000. 10.1371/journal.pbio.1002000 25405475 PMC4236020

[B61] Li A. Saito M. Chuang J.-Z. Tseng Y.-Y. Dedesma C. Tomizawa K. (2011). Ciliary transition zone activation of phosphorylated Tctex-1 controls ciliary resorption. S-phase entry and fate of neural progenitors. *Nat. Cell Biol.* 13 402–411. 10.1038/ncb2218 21394082 PMC4018803

[B62] Li Y. Muffat J. Omer A. Bosch I. Lancaster M. A. Sur M. (2017). Induction of Expansion and Folding in Human Cerebral Organoids. *Cell. Stem Cell* 20 385–396. 10.1016/j.stem.2016.11.017 28041895 PMC6461394

[B63] Li Z. Tyler W. A. Zeldich E. Baró G. S. Okamoto M. Gao T. (2020). Transcriptional priming as a conserved mechanism of lineage diversification in the developing mouse and human neocortex. *Sci. Adv.* 6:eabd2068. 10.1126/sciadv.abd2068 33158872 PMC7673705

[B64] Llinares-Benadero C. Borrell V. (2019). Deconstructing cortical folding: genetic, cellular and mechanical determinants. *Nat. Rev. Neurosci.* 20 161–176. 10.1038/s41583-018-0112-2 30610227

[B65] Lui J. H. Hansen D. V. Kriegstein A. R. (2011). Development and Evolution of the Human Neocortex. *Cell* 146:332 10.1016/j.cell.2011.07.005 PMC3610574 21729779

[B66] Marchand A. Schwartz C. (2019). Perineuronal net expression in the brain of a hibernating mammal. *Brain Struct. Funct.* 225 45–56. 10.1007/s00429-019-01983-w 31748912

[B67] Marino L. (2007). Cetacean brains: how aquatic are they? *Anat. Rec.* 290 694–700. 10.1002/ar.20530 17516433

[B68] Matsumoto N. Tanaka S. Horiike T. Shinmyo Y. Kawasaki H. (2020). A discrete subtype of neural progenitor crucial for cortical folding in the gyrencephalic mammalian brain. *eLife* 9:e54873. 10.7554/elife.54873 32312384 PMC7173966

[B69] Mauney S. A. Pietersen C. Y. Sonntag K. Woo T. W. (2015). Differentiation of oligodendrocyte precursors is impaired in the prefrontal cortex in schizophrenia. *Schizoph. Res.* 169 374–380. 10.1016/j.schres.2015.10.042 26585218 PMC4681621

[B70] Mignone F. Pesole G. (2011). mRNA U^∗^ntranslated Regions (UTRs). *ELS* 10.1002/9780470015902.a0005009.pub2

[B71] Monda J. K. Cheeseman I. M. (2018). Nde1 promotes diverse dynein functions through differential interactions and exhibits an isoform-specific proteasome association. *Mol. Biol. Cell* 29 2336–2345. 10.1091/mbc.e18-07-0418 30024347 PMC6249811

[B72] Moor C. H. Meijer H. Lissenden S. (2005). Mechanisms of translational control by the 3’ UTR in development and differentiation. *Semin. Cell Dev. Biol.* 16 49–58. 10.1016/j.semcdb.2004.11.007 15659339

[B73] Mora-Bermúdez F. Badsha F. Kanton S. Camp J. G. Vernot B. Köhler K. (2016). Differences and similarities between human and chimpanzee neural progenitors during cerebral cortex development. *eLife* 5:e18683. 10.7554/elife.18683 27669147 PMC5110243

[B74] Morawski M. Brückner G. Jäger C. Seeger G. Künzle H. Arendt T. (2010). Aggrecan-based extracellular matrix shows unique cortical features and conserved subcortical principles of mammalian brain organization in the Madagascan lesser hedgehog tenrec (Echinops telfairi Martin, 1838). *Neuroscience* 165 831–849. 10.1016/j.neuroscience.2009.08.018 19682554

[B75] Morris N. R. (1975). Mitotic mutants of Aspergillus nidulans. *Genet. Res.* 26 237–254. 10.1017/s0016672300016049 773766

[B76] Morris N. R. (2000). Nuclear Migration. *J. Cell Biol.* 148 1097–1102. 10.1083/jcb.148.6.1097 10725321 PMC2174304

[B77] Morris N. R. Xiang X. Beckwith S. M. (1995). Nuclear migration advances in fungi. *Trends Cell Biol.* 5 278–282. 10.1016/s0962-8924(00)89039-x 14732112

[B78] Mosca S. Raponi M. Meneghello A. Buratti E. Woods C. G. Baralle D. (2017). Human NDE1 splicing and mammalian brain development. *Sci. Rep.* 7:43504. 10.1038/srep43504 28266585 PMC5339911

[B79] Mota B. Herculano-Houzel S. (2015). Cortical folding scales universally with surface area and thickness, not number of neurons. *Science* 349 74–77. 10.1126/science.aaa9101 26138976

[B80] Muchnik S. K. Lorente-Galdos B. Santpere G. Sestan N. (2019). Modeling the Evolution of Human Brain Development Using Organoids. *Cell* 179 1250–1253. 10.1016/j.cell.2019.10.041 31778651 PMC7034679

[B81] Murciano A. Zamora J. Lopezsanchez J. Frade J. (2002). Interkinetic Nuclear Movement May Provide Spatial Clues to the Regulation of Neurogenesis. *Mol. Cell. Neurosci.* 21 285–300. 10.1006/mcne.2002.1174 12401448

[B82] Nadarajah B. (2003). Neuronal migration in the developing cerebral cortex: observations based on real-time imaging. *Cereb. Cortex* 13 607–611. 10.1093/cercor/13.6.607 12764035

[B83] Noctor S. Martínez-Cerdeño V. Ivic L. Kriegstein A. R. (2004). Cortical neurons arise in symmetric, and asymmetric division zones, and migrate through specific phases. *Nat. Neurosci.* 7 136–144. 10.1038/nn1172 14703572

[B84] Okano H. Sasaki E. Yamamori T. Iriki A. Shimogori T. Yamaguchi Y. (2016). Brain/MINDS: a japanese national brain project for marmoset neuroscience. *Neuron* 92 582–590. 10.1016/j.neuron.2016.10.018 27809998

[B85] Paciorkowski A. R. Keppler-Noreuil K. Robinson L. Sullivan C. Sajan S. Christian S. L. (2013). Deletion 16p13.*11* uncoversNDE1mutations on the non-deleted homolog and extends the spectrum of severe microcephaly to include fetal brain disruption. *Am. J. Med. Genet. Part A* 161 1523–1530. 10.1002/ajmg.a.35969 23704059 PMC3689850

[B86] Pan J. Snell W. (2007). The primary cilium: keeper of the key to cell division. *Cell* 129 1255–1257. 10.1016/j.cell.2007.06.018 17604715

[B87] Pawlisz A. S. Mutch C. Wynshaw-Boris A. Chenn A. Walsh C. A. Feng Y. (2008). Lis1–Nde1-dependent neuronal fate control determines cerebral cortical size and lamination. *Hum. Mol. Genet.* 17 2441–2455. 10.1093/hmg/ddn144 18469343 PMC2486443

[B88] Phelps S. M. Young L. J. (2003). Extraordinary diversity in vasopressin (V1a) receptor distributions among wild prairie voles (Microtus ochrogaster): Patterns of variation and covariation. *J. Comp. Neurol.* 466 564–576. 10.1002/cne.10902 14566950

[B89] Pilaz L.-J. Mcmahon J. J. Miller E. E. Lennox A. L. Suzuki A. Salmon E. (2016). Prolonged mitosis of neural progenitors alters cell fate in the developing brain. *Neuron* 89 83–99. 10.1016/j.neuron.2015.12.007 26748089 PMC4706996

[B90] Pollen A. A. Bhaduri A. Andrews M. G. Nowakowski T. J. Meyerson O. S. Mostajo-Radji M. A. (2018). Establishing cerebral organoids as models of human-specific brain evolution. *Cell* 176 743.e17–756.e17. 10.1101/500934 PMC6544371 30735633

[B91] Porteous D. Millar K. (2009). How DISC1 regulates postnatal brain development: girdin gets in on the AKT. *Neuron* 63 711–713. 10.1016/j.neuron.2009.09.017 19778497

[B92] Raghanti M. A. Wicinski B. Meierovich R. Warda T. Dickstein D. L. Reidenberg J. S. (2018). A comparison of the cortical structure of the bowhead whale (*Balaena mysticetus*), a basal Mysticete, with other Cetaceans. *Anat. Rec.* 302 745–760. 10.1002/ar.23991 30332717

[B93] Reillo I. Romero C. D. García-Cabezas M. Á Borrell V. (2010). A role for intermediate radial glia in the tangential expansion of the mammalian cerebral cortex. *Cereb. Cortex* 21 1674–1694. 10.1093/cercor/bhq238 21127018

[B94] Reiner O. Sapir T. Gerlitz G. (2011). Interkinetic Nuclear Movement in the Ventricular Zone of the Cortex. *J. Mol. Neurosci.* 46 516–526. 10.1007/s12031-011-9633-0 21881827

[B95] Ronan L. Fletcher P. C. (2015). From genes to folds: a review of cortical gyrification theory. *Brain Struct. Funct.* 220 2475–2483. 10.1007/s00429-014-0961-z 25511709 PMC4549381

[B96] Saillour Y. Carion N. Quelin C. Leger P. Boddaert N. Elie C. (2009). LIS1-Related Isolated Lissencephaly. *Arch. Neurol.* 66 1007–1075. 10.1001/archneurol.2009.149 19667223

[B97] Salomoni P. Calegari F. (2010). Cell cycle control of mammalian neural stem cells: putting a speed limit on G1. *Trends Cell Biol.* 20 233–243. 10.1016/j.tcb.2010.01.006 20153966

[B98] Satir P. Christensen S. T. (2008). Structure and function of mammalian cilia. *Histochem. Cell Biol.* 129 687–693. 10.1007/s00418-008-0416-9 18365235 PMC2386530

[B99] Shimizu S. Ishino Y. Tohyama M. Miyata S. (2018). NDE1 positively regulates oligodendrocyte morphological differentiation. *Sci. Rep.* 8:7644. 10.1038/s41598-018-25898-4 29769557 PMC5955916

[B100] Simian M. Bissell M. J. (2016). Organoids: a historical perspective of thinking in three dimensions. *J. Cell Biol.* 216 31–40. 10.1083/jcb.201610056 28031422 PMC5223613

[B101] Simões P. A. Celestino R. Carvalho A. X. Gassmann R. (2017). NudE regulates dynein at kinetochores but is dispensable for other dynein functions in the C. *elegans early embryo*. *J. Cell Sci.* 131:jcs212159. 10.1242/jcs.212159 29192061 PMC5818066

[B102] Soares D. C. Bradshaw N. J. Zou J. Kennaway C. K. Hamilton R. S. Chen Z. A. (2012). The mitosis and neurodevelopment proteins NDE1 and NDEL1 form dimers, tetramers, and polymers with a folded back structure in solution. *J. Biol. Chem.* 287 32381–32393. 10.1074/jbc.m112.393439 22843697 PMC3463352

[B103] Spear P. C. Erickson C. A. (2012a). Apical movement during interkinetic nuclear migration is a two-step process. *Dev. Biol.* 370 33–41. 10.1016/j.ydbio.2012.06.031 22884563 PMC3935435

[B104] Spear P. C. Erickson C. A. (2012b). Interkinetic nuclear migration: a mysterious process in search of a function. *Dev. Growth Differ.* 54 306–316. 10.1111/j.1440-169x.2012.01342.x 22524603 PMC3357188

[B105] Spocter M. A. Patzke N. Manger P. R. (2017). *Cetacean Brains ?. Reference Module in Neuroscience and Biobehavioral Psychology.* New York, NY: Springer, 10.1016/b978-0-12-809324-5.02175-1

[B106] Stepien B. K. Naumann R. Holtz A. Helppi J. Huttner W. B. Vaid S. (2020). Le^∗^ngthening neurogenic period during neocortical development causes a hallmark of neocortex expansion. *SSRN Electr. J.* 10.2139/ssrn.3565014 32888487

[B107] Strzyz P. J. Lee H. O. Sidhaye J. Weber I. P. Leung L. C. Norden C. (2015). Interkinetic Nuclear Migration Is Centrosome Independent and Ensures Apical Cell Division to Maintain Tissue Integrity. *Dev. Cell* 32 203–219. 10.1016/j.devcel.2014.12.001 25600237

[B108] Sullivan W. E. (1982). Neural representation of target distance in auditory cortex of the echolocating bat *Myotis lucifugus*. *J. Neurophysiol.* 48 1011–1032. 10.1152/jn.1982.48.4.1011 7143030

[B109] Tan L. Bi B. Zhao P. Cai X. Wan C. Shao J. (2017). Severe congenital microcephaly with 16p13.*11* microdeletion combined with NDE1 mutation, a case report and literature review. *BMC Med. Genet.* 18:141. 10.1186/s12881-017-0501-9 29191162 PMC5709987

[B110] Thomson P. A. Malavasi E. L. Grünewald E. Soares D. C. Borkowska M. Millar J. K. (2012). DISC1 genetics, biology and psychiatric illness. *Front. Biol.* 8:7. 10.1007/s11515-012-1254-7 23550053 PMC3580875

[B111] Tong C. K. Han Y.-G. Shah J. K. Obernier K. Guinto C. D. Alvarez-Buylla A. (2014). Primary cilia are required in a unique subpopulation of neural progenitors. *Proc. Natl. Acad. Sci. U.S.A.* 111 12438–12443. 10.1073/pnas.1321425111 25114218 PMC4151724

[B112] Tuoc T. C. Pavlakis E. Tylkowski M. A. Stoykova A. (2014). Control of cerebral size and thickness. *Cell. Mol. Life Sci.* 71 3199–3218. 10.1007/s00018-014-1590-7 24614969 PMC11113230

[B113] Ueno M. Katayama K. Yamauchi H. Nakayama H. Doi K. (2006). Cell cycle progression is required for nuclear migration of neural progenitor cells. *Brain Res.* 1088 57–67. 10.1016/j.brainres.2006.03.042 16650835

[B114] Vergnolle M. A. Taylor S. S. (2007). Cenp-F Links Kinetochores to Ndel1/Nde1/Lis1/Dynein Microtubule Motor Complexes. *Curr. Biol.* 17 1173–1179. 10.1016/j.cub.2007.05.077 17600710

[B115] Viot G. Sonigo P. Simon I. Simon-Bouy B. Chadeyron F. Beldjord C. (2004). Neocortical neuronal arrangement in LIS1 and DCX lissencephaly may be different. *Am. J. Med. Genet.* 126A 123–128. 10.1002/ajmg.a.20569 15057976

[B116] Wainman A. Creque J. Williams B. Williams E. V. Bonaccorsi S. Gatti M. (2009). Roles of the Drosophila NudE protein in kinetochore function and centrosome migration. *J. Cell Sci.* 122 1747–1758. 10.1242/jcs.041798 19417004 PMC2684831

[B117] Wang X. Tsai J.-W. Lamonica B. Kriegstein A. R. (2011). A new subtype of progenitor cell in the mouse embryonic neocortex. *Nat. Neurosci.* 14 555–561. 10.1038/nn.2807 21478886 PMC3083489

[B118] Wynshaw-Boris A. (2007). Lissencephaly and LIS1: insights into the molecular mechanisms of neuronal migration and development. *Clin. Genet.* 72 296–304. 10.1111/j.1399-0004.2007.00888.x 17850624

[B119] Xiang X. (2018). Nuclear movement in fungi. *Semin. Cell Dev. Biol.* 82 3–16. 10.1016/j.semcdb.2017.10.024 29241689 PMC5995604

[B120] Xiang X. Morris N. R. (1999). Hyphal tip growth and nuclear migration. *Curr. Opin. Microbiol.* 2 636–640. 10.1016/s1369-5274(99)00034-x 10607624

[B121] Xiao J. Levitt J. Buffenstein R. (2006). A stereotaxic atlas of the brain of the naked mole-rat (Heterocephalus glaber). *Neuroscience* 141 1415–1435. 10.1016/j.neuroscience.2006.03.077 16793211

[B122] Zilles K. Armstrong E. Schleicher A. (1988). The human pattern of gyrification in the cerebral cortex. *Anat. Embryol.* 179 173–179. 10.1007/BF00304699 3232854

[B123] Zilles K. Palomero-Gallagher N. Amunts K. (2013). Development of cortical folding during evolution and ontogeny. *Trends Neurosci.* 36 275–284. 10.1016/j.tins.2013.01.006 23415112

[B124] Żyłkiewicz E. Kijañska M. Choi W. Derewenda U. Derewenda Z. S. Stukenberg P. T. (2011). The N-terminal coiled-coil of Ndel1 is a regulated scaffold that recruits LIS1 to dynein. *J. Cell Biol.* 192 433–445. 10.1083/jcb.201011142 21282465 PMC3101096

