# Supplementary material for: Role of NDE1 in the Development and Evolution of the Gyrified Cortex
Source: Front Neurosci. 2020 Dec 18;14:617513. doi: 10.3389/fnins.2020.617513 (PMC7775536; doi:10.3389/fnins.2020.617513)
Supplement: Supplementary file 1 [file Data_Sheet_1.docx]

>NP_001137451.1 nuclear distribution protein nudE homolog 1 [Homo sapiens]

MEDSGKTFSSEEEEANYWKDLAMTYKQRAENTQEELREFQEGSREYEAELETQLQQIETRNRDLLSENNRLRMELETIKEKFEVQHSEGYRQISALEDDLAQTKAIKDQLQKYIRELEQANDDLERAKRATIMSLEDFEQRLNQAIERNAFLESELDEKENLLESVQRLKDEARDLRQELAVQQKQEKPRTPMPSSVEAERTDTAVQATGSVPSTPIAHRGPSSSLNTPGSFRRGLDDSTGGTPLTPAARISALNIVGDLLRKVGALESKLASCRNLVYDQSPNRTGGPASGRSSKNRDGGERRPSSTSVPLGDKGLDTSCRWLSKSTTRSSSSC

>NP_001006169.1 nuclear distribution protein nudE homolog 1 [Gallus gallus]

MEDSEEHHFSSVEEETRYWKELAMKYKQCAENIQEELCEFQEGSREYEAELETQLQQTESRNRDLLSENN

RLRIELESVKEKFEMQHSEWYRQVSALEDDLAQTKAIKDQLQKYIRELEQANDDLERAKRAAIMSLEDFE

QRLNQAIERNAFLESELDEKENLLESVQRLKDEARDLRQELAVQQKQEKPKTPMRTSLETERTDTAVQAS

LSLPSTPSLHRAPNINIPTPATFRRGFEDSYCATPLTPAARISALNIMGDLLRKVGALESKLASCRNFVY

DQSPDRTTVSMYMNRDALETRMSPHQPLCDTGLVKRLEFGTRPSSTPGPMSHPSQSVVKMLL

>NP_075806.2 nuclear distribution protein nudE homolog 1 isoform a [Mus musculus]

MEDSGKTFESEEEETNYWRDLAMTYKQRAENTQEELREFQEGSREYEAELEAQLQQIETRNRDLLSENNR

LRMELESVKEKFEMQHSEGYRQISALEDDLAQTKAIKDQLQKYIRELEQANDDLERAKRATIMSLEDFEQ

RLNQAIERNAFLESELDEKENLLESVQRLKDEARDLRQELAVQQKQDKPRTPMPGSGQAKRTDMAVQATG

SVPSTPVAHRGPSSGLNTPGMFRRGLDSSTSGTPLTPAARISALNIVGDLLRKVGALESKLASCRNFMYD

QSPSRTSGPASGRGTKNRDGVDRRPGSTSVGDKGSGKRLEFGKPASEPASPALPSAQGVVKLLL

>NP_001239101.1 nuclear distribution protein nudE homolog 1 [Canis lupus familiaris]

MEDSGKTFSSEEEEANYWKDLAMTYKQRAENTQEELREFQEGSREYEAELETQLQQIETRNRDLLSENNR

LRMELETIKEKFETQHSEGYRQISALEDDLAQTKAIKDQLQKYIRELEQANDDLERAKRATIMSLEDFEQ

RLNQAIERNAFLESELDEKENLLESVQRLKDEARDLRQELAVQQKQEKPRTPMPSSVEAERTDTAVQATG

SVPSTPIAHRGPSTSLNTPGTFRRGLDDSMGGTPLTPAARISALNIVGDLLRKVGALESKLASCRNFVYD

QSPNRTSGPASGRGSKHRDGSERQPSSTGVPLGDKGLDTSCRWLSKSTTRSSSSC

>XP_008123664.1 PREDICTED: LOW QUALITY PROTEIN: nuclear distribution protein nudE homolog 1 [Anolis carolinensis]

MNDVERKPFGSMEEERSYWKELAAKYKECADNAQEELREFQEGSREYEAELEMQLQQMETRNRDLLSEXH

RLRAEIEAVKEKITSQHSEGYHRISALEEELAQTKAIKDQLQKYIRELEQSNDDLERAKRATIMSLEDFE

QRLNQAIERNAFLESELDEKETLLESVQRLKDEARDLRQELAVQQKQEKPKALGESPSEAASVPSTPLTR

RTPGLGVTLSGGTFRRGLEESSNSATPLTPAARISALNIVGDLLRKVGALESKLASCRNFVYDQAPYYSR

SPLAASGPHGWGPRPVRLTGPAVLPPQGVVQMLL

>XP_002127520.2 nuclear distribution protein nudE homolog 1 [Ciona intestinalis]

MAELQSEPPAFANAEEKAAYWEGEAKRFEEMTLEVQLELQEFRECSEEIEKELEAQLEQHEKQTKDLRQQ

NTTLTFEYESLKERYEKQQTERFRQVSDLQTEISKLTVTNGELTKDVRELEQRNDDLERTNRVIIVSLDD

FEQRLNQALERNAFLESELDEKDSLSVTVQRLRDEARDLHHELSVRQRKVSESGKHDERSPLPSPQDKVP

TPTTGVPTTPLATSKKLIFNGVTTSHAPPSPTNPDPHKIHQKHVLHNGVDKTPLPPSARISALNIVGDLL

RKFGALESKLASCRNFVHEQPPWQSKRVNQIAPKSSETPHEGSTTDSSKNSSVKIRV

>NP_001093791.1 nuclear distribution protein nudE homolog 1 [Bos taurus]

MADSGKTFSSEEEEANYWKDLAMTYKQRAENTQEELREFQEESREYEAELETQLQQTESKNRDLLMENNH

LRMELETIKEKFKTQHSEGYRQISALEDDLAQTKAIKDKLQKYIRELEQANDDLERAKRATIMSLEDFEQ

RLNQAIERNAFLESELDEKENLLESVQRLKDEARDLRQELAVQQKQEKPRTPMPSSVDAERTDTAVQATG

SVPSTPIAHRGPSSSLNTPGTFRRGLDDSTGGTPLTPAARISALNIVGDLLRKVGALESKLASCRNFVYD

QSPGRASGPASGRGSKNRDSVDRRPGGSNVPLGDKGLGKRLEFGKPSSNVSSPSLPSAQGVVKMLL

>XP_016878838.1 nuclear distribution protein nudE homolog 1 isoform X2 [Homo sapiens]

MEDSGKTFSSEEEEANYWKDLAMTYKQRAENTQEELREFQEGSREYEAELETQLQQIETRNRDLLSENNR

LRMELETIKEKFEVQHSEGYRQISALEDDLAQTKAIKDQLQKYIRELEQANDDLERAKRATIMSLEDFEQ

RLNQAIERNAFLESELDEKENLLESVQRLKDEARDLRQELAVQQKQEKPRTPMPSSVEAERTDTAVQATG

SVPSTPIAHRGPSSSLNTPGSFRRGLDDSTGGTPLTPAARISALNIVGDLLRKVGALESKLASCRNLVYD

QSPNRTGGPASGRSSKNRDGGERRPSSTSVPLGDKGLGKRLEFGKPPSHMSSSPLPSAQGVVKMLL

>NP_001137451.1 nuclear distribution protein nudE homolog 1 [Homo sapiens]

MEDSGKTFSSEEEEANYWKDLAMTYKQRAENTQEELREFQEGSREYEAELETQLQQIETRNRDLLSENNR

LRMELETIKEKFEVQHSEGYRQISALEDDLAQTKAIKDQLQKYIRELEQANDDLERAKRATIMSLEDFEQ

RLNQAIERNAFLESELDEKENLLESVQRLKDEARDLRQELAVQQKQEKPRTPMPSSVEAERTDTAVQATG

SVPSTPIAHRGPSSSLNTPGSFRRGLDDSTGGTPLTPAARISALNIVGDLLRKVGALESKLASCRNLVYD

QSPNRTGGPASGRSSKNRDGGERRPSSTSVPLGDKGLDTSCRWLSKSTTRSSSSC

>NP_001007996.1 nuclear distribution protein nudE homolog 1 [Xenopus tropicalis]

MDDLENNIFNSVEEEIIYWKSVAMKYKTCSEEAQQELQEFQEASREYEAELEAQLQQIEGRNRDLFSENN

RLRMELDGIKEKYEEQHSENYVQICSLEGDLSQTKAVRDQLQKYIRELEQANDDLERAKRATIMSLEDFE

QRLNQAIERNAFLESELDEKENLLESVQRLKDEARDLRQELAVQQKQEKPKSNMGSPETERMDTSVKASV

AIPSAPLTPLSQRGCASTLTSPLSFRTSLDDGYSGTPLTPCARISALNIVGDLLRKVGALESKLASCRNF

VHEQSPNRPLTSVSARMNKTREGIENRLSMASGSSVEKGLIKRLEFGSLPSNTPVQGMHSPQGVVKMII

>NP_001025374.1 nuclear distribution protein nudE homolog 1 [Danio rerio]

MSDPEPASFDSVEQERDYWKEQADKCKQRAEEAQEELQEFQQMSRDYEVELETELKQCDARNRELLTANN

RLRMELENYKEKYETQHSEAVRQISTLERDLAETTAIKDQLHKYIRELEQSNDDLERAKRATIMSLEDFE

QRMNHVIERNAFLESELDEKENLLESVQRLKDEARDLRQELAVQQKQERKPSISLAKDAEKPEATPARPS

SVVNSALPSLLATPSRPPGSGSAFNTPSASYSRIEGLTGTPLTTSARISALDIVGELLRKVGNLESKLAS

CRELHIPEKTSNRALIGQSSPSIARETPEIPSNTNGLYDKGMVKRLDFGTGSKIML

>NP_001097568.1 nudE, isoform B [Drosophila melanogaster]

MESPPMFNSVEDECRYWKERSKQYHKEWTDVKQEYDEFVEQSREMEIEMDATLDQKQSIIKDLTAKLTMF

ERENESLKLKLESHGIDMSNMEKQLETVKKDRDTMKVYLRQLEQKNDDLERAHRILNESIENFEKMLDQA

YEKNALLELEVDEKGLLQEKLQRLMDETRDLKQELNVKSRFTPVVNGTSVPTANDTNTVNSSMNSSASLP

NGIVANGELVKHDNAVATRATSVSVNALNGSLVNRNEYNQQHSLKNPENQINGNSMNPSSRTTALNIVAD

MLRKLNAMETKLKTYRENGQPMPQRHRSSQQSTHPALAGLPCFSLDAE

>XP_663729.1 hypothetical protein AN6125.2 [Aspergillus nidulans FGSC A4]

MPSADEPSSTRTNGTSSRSDQLAYYKKQYEQLESELADFQASSRELEAELEKEIEASEKRERQLKEKVDN

LRYEVEEWKSKYKQSKSEASTAQNALQKEITSLRDANLTLQLKLRDTEVANDDYERQARHTTSSLEDMES

KYNQALEREVLLDMEYKQGEQERESLRIENQRLRDELNDLKIETEIVQERLRNNNRRRRPAPLGRSPSTP

HTPEIFDRSPGESTVSSPLFSTPPTKLSLTLASATATPPSPPMSETSSSMRKSLTAASGFPLQKASASES

FGTRSLYGNRPQRFQAHSRATSYAFSNGRSTPSATTTRPSLPKANNTTANRPSGIPKSGSLHQIRGLIGK

MQKLEERVQSAKSKLPPPSETASRASSRAGSMLDASPGAATIAMRRDTRKRLSGSSFSSSVRDGDGAPSY

VTSSRPSYGTRTQGDSRPSSRTSFSSSLSHSTHPSVTPSNRPESRQSRTKTPLGHYSTNPTTESRRPRSS

LSNPAGQSTPINGMTYIDEDEDLAEQFNMRATISSTRPTRLPSFSNPAFSTPTGLKKRSTSGMSGIPAPR

TLRRGNTMGPPKTKPKPVAGDLGETF

>XP_020941825.1 nuclear distribution protein nudE homolog 1 isoform X1 [Sus scrofa]

MEDSGKTFSSEEEEANYWKDLAMTYKQRAENTQEELREFQEGSREYEAELETQLQQIETRNRDLLSENNR

LRMELETIKEKFETQHSEGYRQISALEDDLAQTKAIKDQLQKYIRELEQANDDLERAKRATIMSLEDFEQ

RLNQAIERNAFLESELDEKENLLESVQRLKDEARDLRQELAVQQKQEKPRTPMPSSVEAERTDTAVQATG

SVPSTPIVHRGPSSSLNTPGTFRRGLDDSTGGTPLTPAARISALNIVGDLLRKVGALESKLASCRNFVYD

QSPNRTSGPGSGRGSKSRDGGDRRPGSTSVPLGDKGLGKRLEFGKPPSNISSPSLPSAQGVVKMLL

>XP_003377220.1 nuclear distribution protein NudE protein [Trichinella spiralis]

MSDIITLKNEVNHWKSVAESFKNNFATLKEEYNDFQAESRELESELEAQLDQTEKKNSDLMRHNQQLKME

CNGLRTKLECLQNENRKQVTMLEEELNRLRVERDELHRYVRELEQVNDHLERAHREAAVSLEDYDRRLNE

AIERNALLESELDEKDALRAHIQRLKEETRDLKQELDVKGRIEQTAYVKNSRMSESHIPNGDVKRVSLRK

PLERSTSTGEVNRCSATTSRLTAFTILTDLWRRAGFHYKSLNKVSFNGGVLVDSSNSSSSSERTKSKK
